# The temporal protection and declining health of the COVID-19 vaccinated in England: A 26-month comparison of the mortality involving and not involving COVID-19 among vaccinated vs. unvaccinated

**DOI:** 10.12688/f1000research.160980.1

**Published:** 2025-01-27

**Authors:** Jarle Aarstad

**Affiliations:** 1 Western Norway University of Applied Sciences, Bergen, Norway

**Keywords:** COVID-19 vaccination; all-cause mortality; mortality involving COVID-19; mortality not involving COVID-19; excess mortality.

## Abstract

**Background:**

Comparing non-randomized groups, such as COVID-19 vaccinated and unvaccinated, even in the presence of seemingly relevant control variables, is challenging, but in this study, using English data, I show an achievable approach.

**Methods:**

First, I estimated age-standardized all-cause mortality among COVID-19 vaccinated and unvaccinated ten years and older, covering a 26-month period from Apr 21 to May 23. Then, I estimated mortality not involving COVID-19, and finally, I differentiated the calculations.

**Results:**

First, I found that all-cause mortality among COVID-19 unvaccinated was higher than among vaccinated. But as the pattern was similar concerning mortality not involving COVID-19, the discrepancy is attributed mainly to unvaccinated having inferior health at the outset. There was nonetheless significant protection for vaccinated between July 21 and Jan 22. Absent of control variables as a means to compare non-randomized groups, I reached that finding by differentiating all-cause mortality from mortality not involving COVID-19. However, while mortality not involving COVID-19 decreased among unvaccinated compared to the first observation month, it was high among vaccinated, i.e., a relative increase in mortality among vaccinated.

**Conclusions:**

An interpretation is that vaccination, despite temporary protection, increased mortality. Strengthening the interpretation was relatively high mortality among vaccinated not involving COVID-19 counterintuitively following periods of excess mortality. Further strengthening the interpretation was relatively high mortality not involving COVID-19 among vaccinated corresponding with the excess mortality during the same period.

## Introduction

According to the UK Office for National Statistics, ^1^ rates for COVID-19 unvaccinated adults in England “were higher for Black Caribbean, Black African and White Other ethnic groups. Rates were also higher for those living in deprived areas, who have never worked or are long-term unemployed, who are limited a lot by a disability, … or who are male.” The statement indicates that unvaccinated have inferior health at the outset than vaccinated, inducing biased comparisons as the groups are not randomly assigned. Therefore, matching, balancing, ^2^ or controlling for potential confounders, e.g., ethnicity, employment-, disability-, socioeconomic status, and gender may debias the results. ^3^ However, variables accounting for potentially confounding effects are often unavailable or unknown, and including those available but unknowingly improper can increase bias. ^4^ In line with the reasoning, York (Ref. 4, p. 675) showed that “unless *all* potential confounding factors are included in an analysis (which is unlikely to be achievable with most real-world data-sets), adding control variables to a model in many circumstances can make estimated effects … less accurate.”

Due to the addressed issues, comparing non-randomized groups, such as COVID-19 vaccinated and unvaccinated, even in the presence of seemingly relevant control variables, is challenging, but in this study, using English data, ^5^ I show an achievable approach. Initially, I (i) estimated age-standardized all-cause mortality among COVID-19 vaccinated and unvaccinated ten years and older, covering a 26-month period from Apr 21 to May 23. Then, I (ii) estimated mortality not involving COVID-19, and finally, using xlincom, ^6^ an extension of Stata’s ^7^ lincom algorithm, I differentiated the results of (i) and (ii). As all-cause mortality estimates include cases involving COVID-19, I show that differentiating those from estimates not involving COVID-19 cases can identify potentially genuine effects of vaccination between populations with different health statuses at the outset.

Research has indicated that COVID-19 vaccination can prevent mortality, ^8– 11^ but the effect declines. ^12^ Applying my approach to the English data, I show how the results converge with the other studies.

## Methods

I used publicly available data on the population in England ten years and older provided by the UK Office for National Statistics ^5^ for this study. Particularly, I analyzed their data on monthly age-standardized mortality by vaccination status and classification of deaths involving and not involving COVID-19. ^13, 14^ The period for which data were available and included in this study was between Apr 21 and May 23, 26 months.

To exemplify, in Apr 21, the age-standardized all-cause mortality rate among “ever vaccinated”, defined as vaccinated in this study, was 812.7 per 100,000 person-years, which were 2,124,523 that month. ^5^ The expression (812.7/100,000)*2,124,523 gives 17,266 estimated deaths in an estimated population of 25,494,276, which was reached by multiplying 2,124,523 by 12. I.e., the age-standardized all-cause mortality probability among vaccinated in Apr 21 was 17,266 divided by 25,494,276, taking the value of.068 percent. Similar estimations of all-cause mortality, mortality involving and not involving COVID-19, were carried out each month for vaccinated and unvaccinated.

The data were applied in logistic regressions using Stata 17, ^7^ followed by probability estimations using the margins effect command ^15^ and estimations with odds ratios (ORs). Concerning OR estimations, I particularly explain and show below how the xlincom algorithm, ^6^ an extension of Stata’s ^7^ lincom algorithm, was used to differentiate log odds (the logarithm of the ORs) estimates. Also, I explain the substantial interpretation of differentiated estimates.

## Results

**Figure 1.**
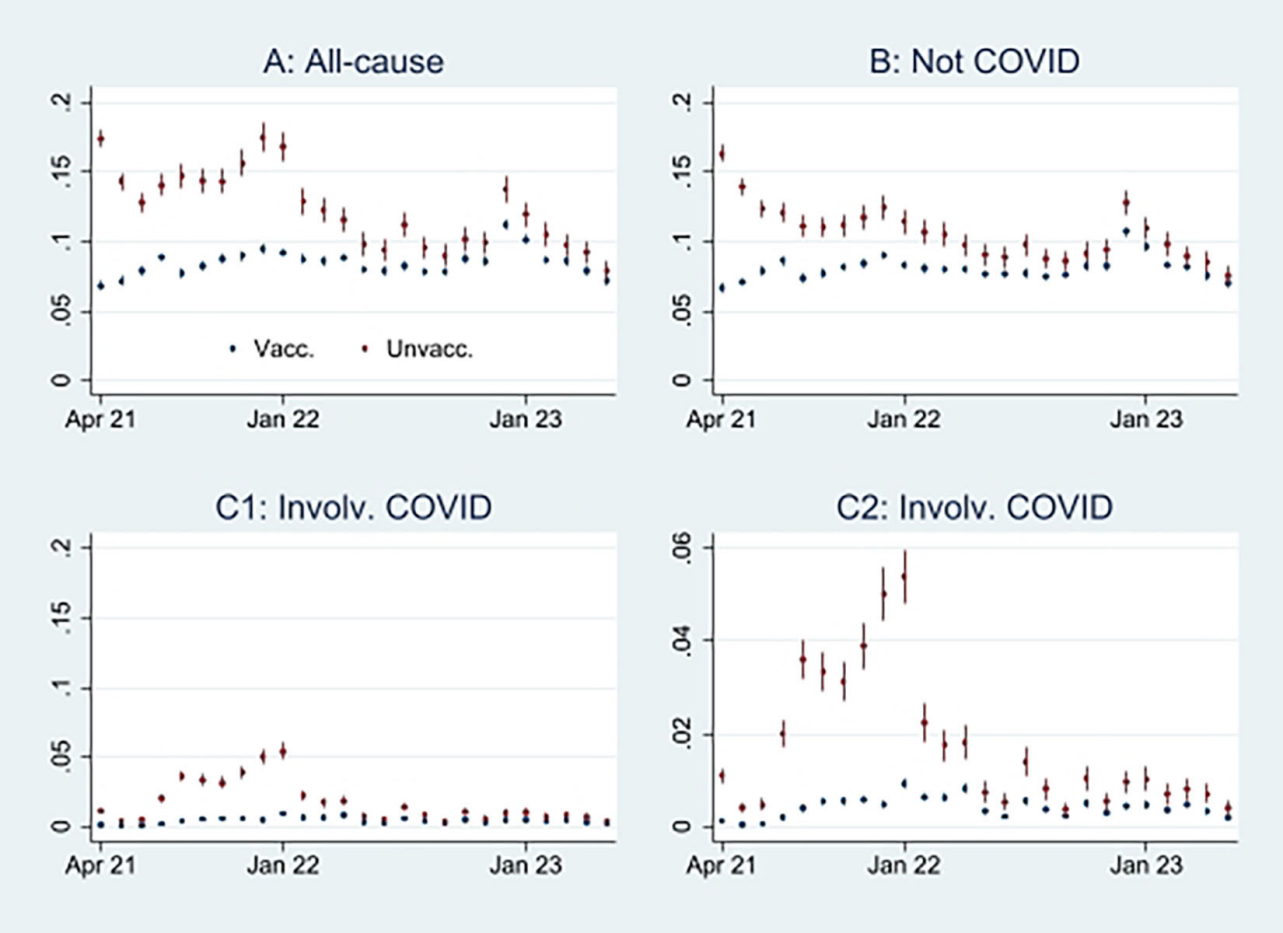
Monthly mortality probabilities in percent with 95% CIs.

**Figure 2.**
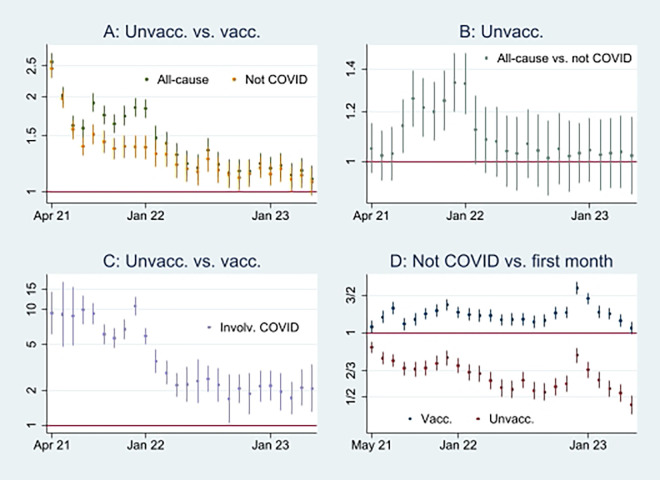
Monthly ORs of mortality with 95% CIs.

Here, I first present the empirical results of age-standardized mortality probabilities among vaccinated and unvaccinated ten years and older, shown in Figure 1. Aided by odds ratios (ORs) calculations shown in Figure 2, I then address the results’ substantial interpretation.

### Initial mortality probability analyses

Figure 1A shows that the all-cause mortality probability, particularly at the beginning of the period, was higher among unvaccinated than vaccinated. The mortality decreased among the unvaccinated, but among the vaccinated, it was relatively stable or had a slight increase. Consequently, the mortality among unvaccinated and vaccinated almost was tangent at the end of the period.

Figure 1B shows that the mortality probability not involving COVID-19 was similar to the all-cause mortality probability ( Figure 1A), except for a lower rate among unvaccinated between the last half of 21 and the beginning of 22. Figures 1C1 and 1C2, identical except for different scaling, show that the mortality probability involving COVID-19 was higher among unvaccinated than vaccinated, particularly between the last part of 21 and the beginning of 22.

An interpretation of Figure 1A can be that the vaccinated had a temporal but declining mortality protection, which aligns with previous research. ^12^ But as the pattern was similar concerning mortality not involving COVID-19 ( Figure 1B), the difference can alternatively be attributed to unvaccinated having inferior health at the outset. ^1^ The reason for the latter assumption is that there is no logical explanation as to how vaccination can prevent mortality that does not involve COVID-19. ^16^

### Odds Ratio analyses

To gain more knowledge about the above issues, Figure 2A shows ORs of all-cause mortality and mortality not involving COVID-19 among unvaccinated compared to vaccinated as a reference group [ 1]. At the beginning of the period, the ORs of mortality among unvaccinated were about 2 and 2,5 compared to vaccinated, and significant (95% CIs). A similar pattern concerning all-cause mortality and mortality not involving COVID-19 indicates that vaccination did not have a preventive effect (as it logically cannot have a preventive effect against mortality not involving COVID-19). However, between the last half of 21 and the beginning of 22, the ORs were higher for all-cause mortality than for mortality not involving COVID-19, which indicates a temporal preventive vaccine effect.

Figure 2B adds further information showing that ORs of all-cause mortality compared to mortality not involving COVID-19 between July 21 and Jan 22 were significant (95% CIs), with most values above 1.2. The results were reached by using Stata’s ^7^ xlincom algorithm ^6^ first to differentiate the log odds (the logarithm of the ORs) of estimates reported in Figure 2A, and next generate the new ORs from the differentiated log odds [ 2]. Accordingly, a conclusion so far is that vaccinated were significantly (CIs 95%) protected between July 21 and Jan 22 [ 3].

### How Odds Ratios and probability analyses indicate declining health among vaccinated

**Figure 3.**
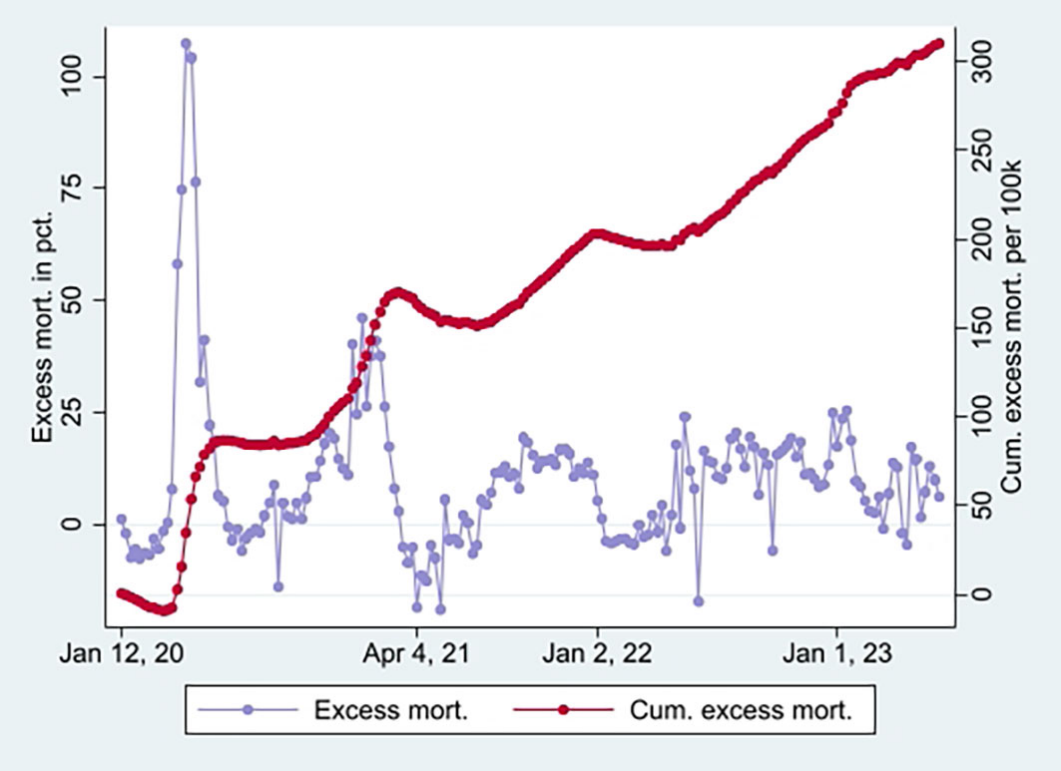
Weekly UK excess mortality in percent and cumulative excess mortality.

Figure 2D shows that while mortality not involving COVID-19 decreased among unvaccinated compared to the first observation month, it was high among vaccinated [ 4]. The results reflect mortality probabilities in Figure 1B, which were almost tangent at the end of the period. Also, they reflect the declining ORs of unvaccinated reported in Figure 2A, taking a non-significant value of a little over 1 at the end (95% CI). Hence, the data show a relatively high and relative increase in mortality not involving COVID-19 among vaccinated. An interpretation is that vaccination, despite temporary protection, increased mortality. ^16^ Strengthening the interpretation was relatively high mortality among vaccinated not involving COVID-19 counterintuitively following periods of excess mortality ( Figure 3) [ 5]. Further strengthening the interpretation was the relatively high mortality rate not involving COVID-19 among the vaccinated corresponding with the excess mortality rate during the same period (ibid.) [ 6].

## Discussion

This study, describing and using an approach to compare non-randomized groups absent of control variables, found that COVID-19 vaccination temporally protected against mortality, which aligns with other research. ^8– 12^ A further interpretation of the data, nonetheless, indicated that COVID-19 vaccination, despite temporary protection, increased mortality. ^16^ It also aligns with other research suggesting that COVID-19 vaccination can have adverse effects ^17– 19^ and increase mortality. ^20^

During the study period, a share of people in the unvaccinated group were transferred to the vaccinated. Assuming they had inferior health status at the outset, it may explain the relative increase (decrease) in mortality among the vaccinated (unvaccinated). However, those who *remained* unvaccinated, on the contrary, had inferior health status at the outset, ^1^ making the above reasoning implausible. Ceteris paribus, one may even oppositely conclude that it would decrease (increase) relative mortality among vaccinated (unvaccinated) [ 7]. Since most elderly candidates had been offered vaccine before Apr 21, ^1, 21^ I nonetheless assume the estimates were not substantially skewed over the study period, as relatively few people die in younger age cohorts.

The study’s validity hinges on non-systematic skewness in classifying false positives concerning mortality involving COVID-19 and false negatives concerning mortality not involving COVID-19. However, I cannot see any substantial reason for substantial skewness in false positives and negatives between vaccinated and unvaccinated, but it may induce some cautiousness when interpreting the data.

This study included those ten years and older. I, therefore, encourage future research to analyze different age cohorts separately to assess how findings may converge or eventually diverge. As this study merely distinguished between those vaccinated and those who were not, I also encourage future research to distinguish between those who received one or more doses and different vaccine types, although it may be methodologically challenging.

## Ethics and consent

Ethical approval and consent were not required.

## Data Availability

UK Office for National Statistics. ^5^ Deaths by vaccination status, England 2023: https://www.ons.gov.uk/peoplepopulationandcommunity/birthsdeathsandmarriages/deaths/datasets/deathsbyvaccinationstatusengland I used the dataset labeled “Deaths occurring between 1 April 2021 and 31 May 2023 edition of this dataset”, Table 1: Unvaccinated and Ever vaccinated. The Methods section explains in detail how I modeled the data.

## Footnotes

^1^
Vertical axes in Figure 2 are log-transformed using the natural logarithm.

^2^
Note that overlapping 95% CIs July 21 in Figure 2A appears inconsistent with significant OR (95% CI) for the same month in Figure 2B, but the issue is discussed by Knol, Pestman and Grobbee. ^22^

^3^
One may attribute the seemingly non-significant vaccine protection from Feb 22 ( Figure 2B) to relatively low mortality involving COVID-19 during that period ( Figures 1C1 and 1C2). The reason for the argument is that the effects in Figure 2B would be absent if the mortality involving COVID-19 were to approach zero (which explains the non-significant effect between Apr and June 21). However, Figure 2C shows that the ORs of mortality among unvaccinated (compared to vaccinated as a reference group) involving COVID-19 were down from about 10 at the beginning to about 2 at the end. It implies that not only was the mortality among unvaccinated not involving COVID-19 relatively low at the end ( Figure 2A), but also the mortality involving COVID-19 ( Figure 2C). If the latter were high, one could argue that low mortality involving COVID-19 is a valid explanation for the non-significant effect from Feb 22 ( Figure 2B) but less so when the ORs of mortality among unvaccinated were also relatively low ( Figure 2C). The ORs in Figure 2C were significant (95% CIs) during the whole period, which can be due to (i) vaccine protection and (ii) unvaccinated having inferior health at the outset. ^1^ However, as the ORs were only about a fifth since Feb 22 compared to the first months, explanation (ii) is more likely during that period.

^4^
The overall pattern among unvaccinated was similar both concerning all-cause mortality and mortality not involving COVID-19. Therefore, one cannot claim that the overall decrease in mortality not involving COVID-19 was due to mortality involving it.

^5^
Assuming that the excess mortality among the unvaccinated segment before Apr 21 was *a* percent, taking a positive value, one may assume that it was *a** *b* percent among the vaccinated segment, where 0< *b*<1. One may assume *b*<1 because the vaccinated segment had relatively good health at the outset, ^1^ and one may assume 0< *b* because there were, nonetheless, people vulnerable to COVID-19 among them. I.e., *a** *b* was lower than *a* but still higher than zero. According to the reasoning, one should expect a decline in mortality among vaccinated during the study period due to previous excess mortality, but not necessarily as marked as observed among unvaccinated. Alternatively, one may argue the opposite as among the vaccinated segment, “some very comorbid patients [in care homes] got vaccine side effects that probably accelerated an already progressing death process” (Ref. 23, p. 3 - my translation from Norwegian).

^6^

Figure 3 shows weekly UK excess mortality in percent and cumulative excess mortality. ^24^ English monthly data ^25^ show similar patterns concerning excess mortality in percent.

^7^
People in England under 70 years old but clinically extremely vulnerable were prioritized vaccination with those aged between 70-74. ^21^ Hence, they were prioritized early.
