# Mortality involving and not involving COVID-19 among vaccinated vs. unvaccinated in England between Apr 21 and May 23

**DOI:** 10.12688/f1000research.160980.2

**Published:** 2025-04-03

**Authors:** Jarle Aarstad

**Affiliations:** 1 Western Norway University of Applied Sciences, Bergen, Norway

**Keywords:** COVID-19 vaccination; all-cause mortality; mortality involving COVID-19; mortality not involving COVID-19; excess mortality.

## Abstract

**Background:**

Comparing non-randomized groups, such as COVID-19 vaccinated and unvaccinated, even in the presence of seemingly relevant control variables, is challenging, but in this study, using English data, I show an achievable approach.

**Methods:**

First, I estimated age-standardized all-cause mortality among vaccinated and unvaccinated ten years and older, covering 26 months from Apr 21 to May 23. Then, I estimated mortality not involving COVID-19, and finally, I differentiated the calculations.

**Results:**

First, I found that all-cause mortality among unvaccinated was higher than among vaccinated. But, as the pattern was similar concerning mortality not involving COVID-19, the discrepancy is attributed mainly to unvaccinated having inferior health at the outset. There was nonetheless significant protection for vaccinated between July 21 and Jan 22. Absent of control variables as a means to compare non-randomized groups, I reached that finding by differentiating all-cause mortality from mortality not involving COVID-19. However, while mortality not involving COVID-19 decreased among unvaccinated compared to the first observation month, it was high among vaccinated, i.e., a relative increase in mortality among vaccinated.

**Conclusions:**

An interpretation is that vaccination, despite temporary protection, increased mortality. Strengthening the interpretation was relatively high mortality among vaccinated not involving COVID-19 counterintuitively following periods of excess mortality. Further strengthening the interpretation was relatively high mortality not involving COVID-19 among vaccinated, corresponding with excess mortality during much of the same period. An implication of the study, which particularly has relevance for future pandemics, is that vaccinated may have a limited time window of protection and can even be exposed to detrimental health consequences. The pattern should be followed up over an extended period in future research. Also, future research should examine different age groups, vaccination types, and the number of doses given.

## Introduction

According to the UK Office for National Statistics, ^1^ rates for COVID-19 unvaccinated adults in England “were higher for Black Caribbean, Black African and White Other ethnic groups. Rates were also higher for those living in deprived areas, who have never worked or are long-term unemployed, who are limited a lot by a disability, … or who are male.” The statement aligns with vaccine hesitancy research ^2, 3^ and further indicates that unvaccinated have inferior health at the outset compared to vaccinated, inducing biased comparisons as the groups are not randomly assigned. Therefore, matching, balancing, ^4^ or controlling for potential confounders, e.g., ethnicity, employment-, disability-, socioeconomic status, and gender, may debias the results. ^5^ However, variables accounting for potentially confounding effects are often unavailable or unknown, and including those available but unknowingly improper can increase bias. ^6^ In line with the reasoning, York (Ref. 6, p. 675) showed that “unless *all* potential confounding factors are included in an analysis (which is unlikely to be achievable with most real-world data-sets), adding control variables to a model in many circumstances can make estimated effects … less accurate.”Norwegian research exemplifies that showing 30% lower all-cause mortality among COVID-vaccinated compared unvaccinated, 18-44 years, and 58% when including control variables. ^7^ The findings are unattributable to a vaccine effect as close to zero young people died of COVID-19 in Norway, ^8^ and illuminate two issues: (i) COVID-19 vaccinated and unvaccinated have different health status at the outset and (ii) including control variables can make estimates less, not more, accurate, both consistent with my outline above.

Hence, I argue there is a research gap concerning valid estimations between non-randomized groups, such as COVID-19 vaccinated and unvaccinated, which is challenging even when including seemingly relevant control variables that can actually deteriorate the results. ^7^ To address the research gap, using English data covering 26 months from Apr 21 to May 23, ^9^ I elaborate an achievable approach by comparing all-cause mortality among COVID-19 vaccinated and unvaccinated with mortality not involving COVID-19. In the Methods section, I explain it in full detail.

The study’s research question is accordingly as follows: Applying the approach addressed above (which I further elaborate on in the Methods section), how do the mortality patterns differ in England from Apr 21 to May23 between COVID-19 vaccinated and unvaccinated? The study’s major contribution is to illustrate how comparing mortality involving and not involving COVID-19 can assess valid estimates between non-randomized groups of vaccinated and unvaccinated.

COVID-19 vaccination has been recommended to most population groups, including people with comorbidities. ^10^

Studies have further indicated that COVID-19 vaccination can prevent mortality, ^11– 17^ but along with research showing that antibody levels were a superior predictor, ^18^ the effect declines, ^19^ and research has even shown “a positive correlation between people fully vaccinated and COVID-19 mortality”. ^20^ Applying my approach to the English data, I particularly contribute to the research on the link between COVID-19 vaccination and mortality, as most previous studies have been carried out in non-randomized contexts and, accordingly, even in the presence of control variables, exposed to challenges concerning validity addressed above.

## Methods

### Sample and data

I used publicly available data on the population in England ten years and older provided by the UK Office for National Statistics ^9^ for this study. Particularly, I applied their data on monthly age-standardized all-cause mortality and mortality not involving COVID-19 by vaccination status, ^21, 22^ and present further details below. The period for which data were available and included in this study was between Apr 21 and May 23, 26 months.

### Measures of variables

The study includes the two effect variables, monthly mortality rates and monthly odds ratios (ORs) of mortality. As noted, I distinguished between all-cause mortality and mortality not involving COVID-19. All-cause mortality implies anybody who died independent of cause. Mortality not involving COVID-19 implies those who died but did *not* have COVID-19 mentioned on the death certificate in terms of ICD10 codes U07.1 (COVID-19, virus identified) or U07.2 (COVID-19, virus not identified).

COVID-19 vaccinated for this study were defined as those having received one or more doses, labeled as “ever vaccinated” in the raw data, and unvaccinated were defined as those not having received any dose. Each month, I classified those who either died of any cause (all-cause mortality) or survived as either COVID-19-vaccinated or unvaccinated. Hence, each month, a person in the data was classified as (i) dead and vaccinated, (ii) alive and vaccinated, (iii) dead and unvaccinated, or (iv) alive and unvaccinated. I made similar classifications concerning mortality not involving COVID-19.

To exemplify how I classified the data, in Apr 21, the age-standardized all-cause mortality rate among “ever vaccinated”, i.e., defined as vaccinated in this study, was 812.7 per 100,000 person-years, which were 2,124,523 that month. ^9^ The expression (812.7/100,000)*2,124,523 gives 17,266 estimated deaths in an estimated population of 25,494,276, which was reached by multiplying 2,124,523 by 12. I.e., the age-standardized all-cause mortality rate per 100,000 vaccinated in Apr 21 was 17,266 divided by 25,494,276 multiplied by 100,000, taking the value of 67.7. Similar estimations of all-cause mortality and mortality not involving COVID-19, were carried out each month for vaccinated and unvaccinated. (In the Notes section, I also present estimations *involving* COVID-19. I.e., estimations excluding mortality not involving COVID-19.) I carry out the exercise, assessing how many died or survived of a population in a given month, vaccinated or unvaccinated, to estimate as statistically correct standard errors as possible using logistic regression.

### Models and data analysis procedure

The data were applied in logistic regressions using Stata 17. ^23^ I used the margin effect command to estimate mortality rates, ^24^ followed by OR estimations.

Initially, I (i) estimated monthly age-standardized all-cause mortality rate per 100,000 among COVID-19 vaccinated and unvaccinated. Then, I (ii) estimated mortality rate not involving COVID-19, and finally, using xlincom, ^25^ an extension of Stata’s ^23^ lincom algorithm, I differentiated the results of (i) and (ii), and presented the results as ORs. Concerning OR estimations, I particularly explain and show in the Results section how the xlincom algorithm was used to differentiate log odds (the logarithm of the ORs) estimates. Also, I explain the substantial interpretation of differentiated estimates.

As all-cause mortality estimates include cases involving COVID-19, I show that differentiating those from estimates not involving COVID-19 cases can identify potentially genuine effects of vaccination between populations with potentially different health statuses at the outset. The following paragraph illuminates my argument.

Assuming a 60% higher all-cause mortality rate among unvaccinated compared to vaccinated, in the absence of other information, can have two explanations: (i) the unvaccinated have inferior health at the outset compared to the vaccinated or (ii) vaccination protects against mortality. In addition, there can be a combination of (i) and (ii). If the mortality not involving COVID-19 is also 60% higher among unvaccinated, explanation (i) has more validity. The reason is that COVID-19 vaccination unlikely protects against mortality not involving COVID-19. ^26^ Conversely if the mortality rate not involving COVID-19 is equal between unvaccinated and vaccinated, explanation (ii) has higher validity. The reason is that there is no other likely explanation than a vaccine effect as to why the all-cause mortality among unvaccinated compared to unvaccinated is higher than the mortality not involving COVID-19. Finally, if the mortality not involving COVID-19 is 20% higher among unvaccinated compared to the vaccinated, a combination of explanations (i) and (ii) has more validity. I.e., 20% higher mortality not involving COVID-19 among unvaccinated can be explained as inferior health status at the outset, while vaccination protection can explain 33% higher mortality among unvaccinated (((1.6/1.2)-1)*100). The explanations hinge on the assumption of non-systematic skewness in classifying false positives concerning mortality involving COVID-19 and false negatives concerning mortality not involving COVID-19, which I address in the Discussion. Further, the explanations hinge on the assumption that the mortality involving COVID-19 is not zero, which I address in Note 3.

## Results

**Figure 1.**
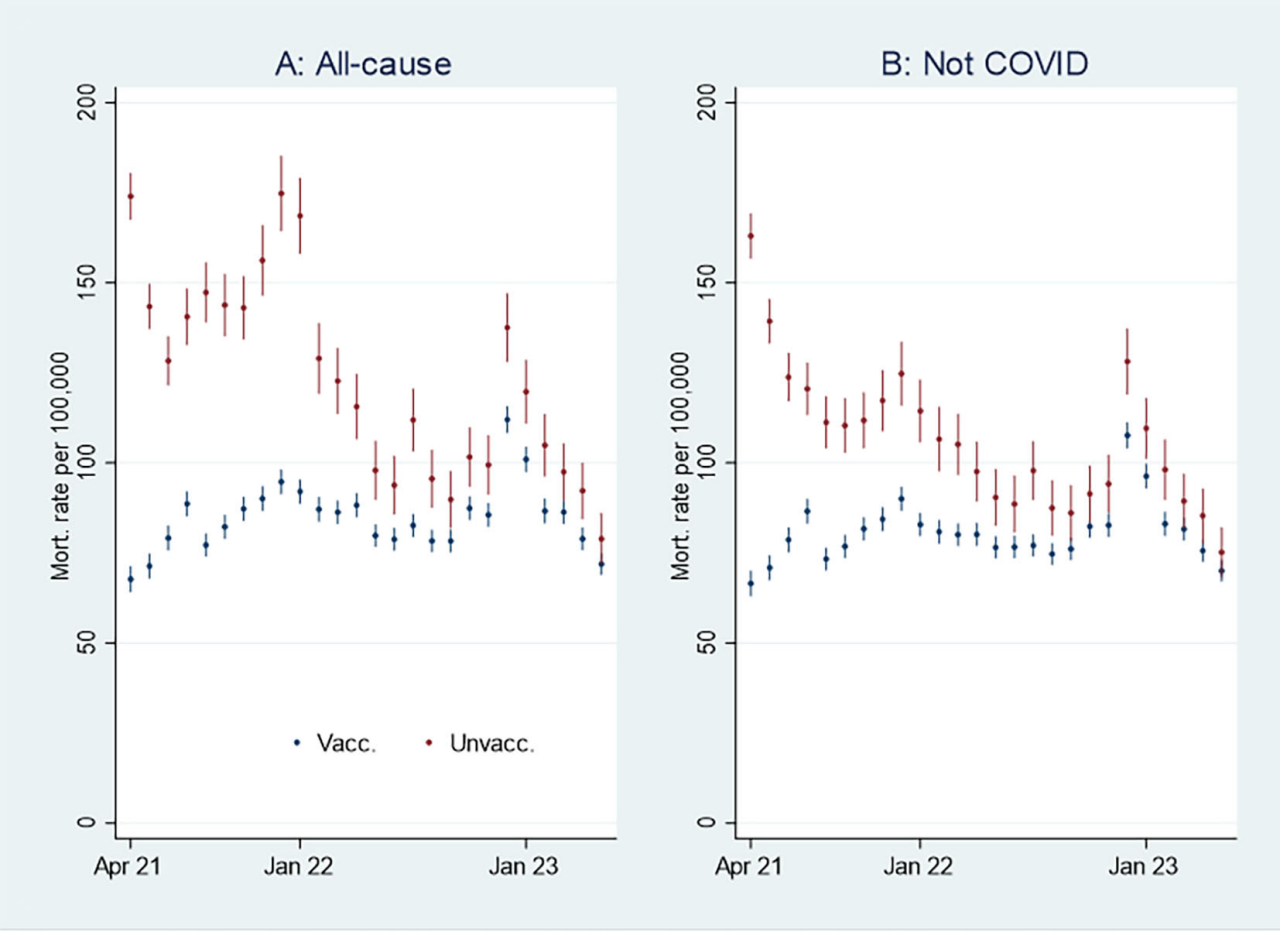
Monthly mortality rates per 100,000 with 95% CIs.

**Figure 2.**
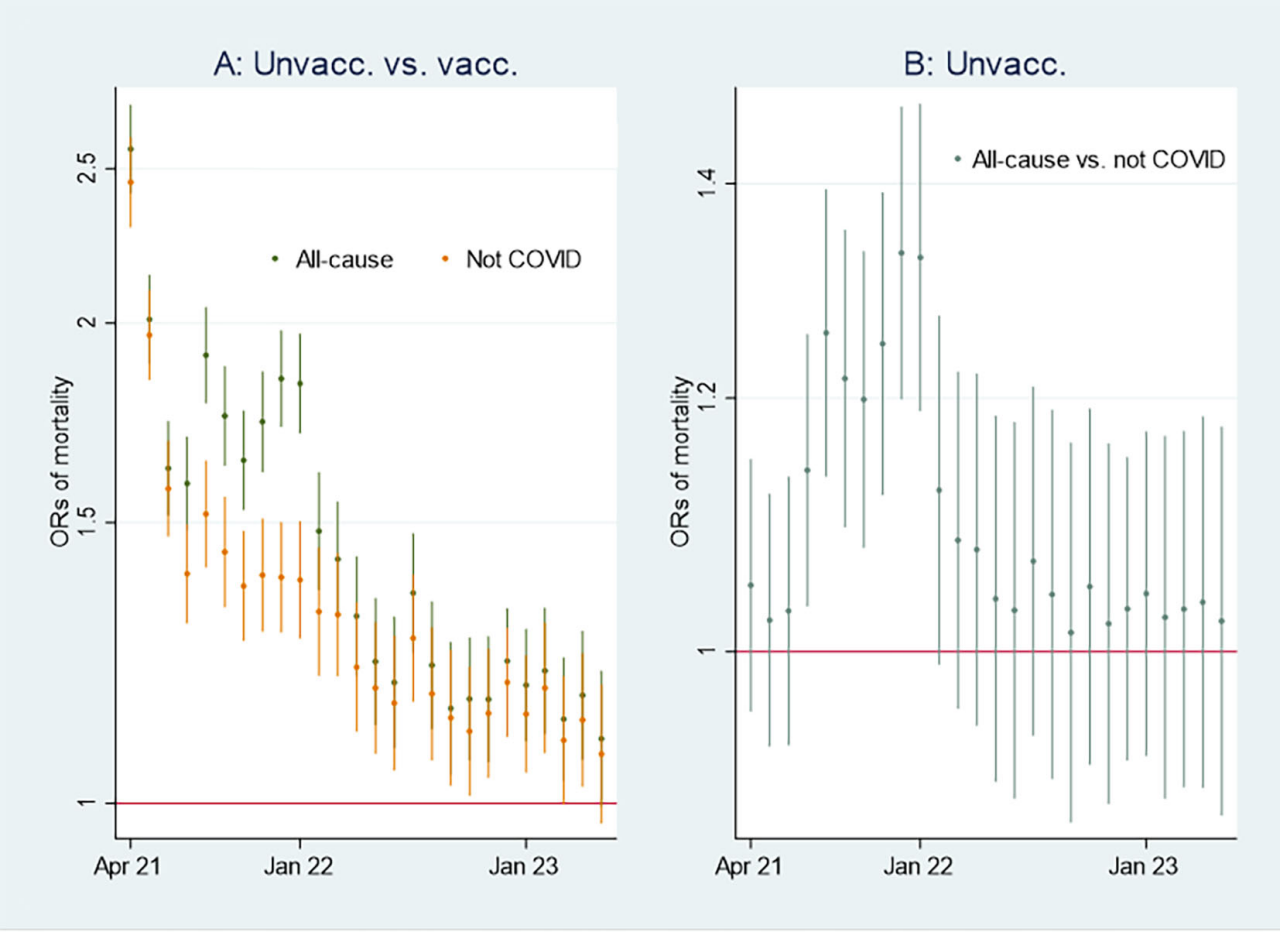
Monthly ORs of mortality with 95% CIs.

I first present the empirical results of age-standardized mortality rates among vaccinated and unvaccinated ten years and older, shown in Figure 1. Aided by odds ratios (ORs) calculations shown in Figure 2, I then address the results’ substantial interpretation.

### Initial mortality rate analyses

Figure 1A shows that the monthly all-cause mortality rate, particularly at the beginning of the period, was higher among unvaccinated than vaccinated. The rate decreased among the unvaccinated, but among the vaccinated, it was relatively stable or had a slight increase. Consequently, the mortality among unvaccinated and vaccinated almost was tangent at the end of the period.

Figure 1B shows that the mortality rate not involving COVID-19 was similar to the all-cause mortality rate ( Figure 1A), except for being lower among unvaccinated between the last half of 21 and the beginning of 22.

An interpretation of Figure 1A can be that the vaccinated had a temporal but declining mortality protection. However, as the pattern was similar concerning mortality not involving COVID-19 ( Figure 1B), the difference can alternatively be attributed to unvaccinated having inferior health at the outset (cf. my explanation at the end of the Methods section, and which I further elaborate on below).

### Odds ratio analyses

To learn more about the above issues, Figure 2A shows ORs of all-cause mortality and mortality not involving COVID-19 among unvaccinated compared to vaccinated as a reference group [ 1]. At the beginning of the period, the ORs of mortality among unvaccinated were about 2 and 2,5 compared to vaccinated, and significant (95% CIs). A similar pattern concerning all-cause mortality and mortality not involving COVID-19 indicates that vaccination did not have a preventive effect (as it logically cannot have a preventive effect against mortality not involving COVID-19, cf. my explanation at the end of the Methods section). However, between the last half of 21 and the beginning of 22, the ORs were higher for all-cause mortality than for mortality not involving COVID-19, which indicates a temporal preventive vaccine effect.

Figure 2B adds further information showing that ORs of all-cause mortality compared to mortality not involving COVID-19 between July 21 and Jan 22 were significant (95% CIs), with most values above 1.2. The results were reached by using Stata’s ^23^ xlincom algorithm ^25^ first to differentiate the log odds (the logarithm of the ORs) of estimates reported in Figure 2A, and next generate the new ORs from the differentiated log odds [ 2]. Accordingly, a conclusion so far is that vaccinated were significantly (CIs 95%) protected between July 21 and Jan 22 [ 3].

### Odds ratios and mortality rate analyses indicate declining health among vaccinated

**Figure 3.**
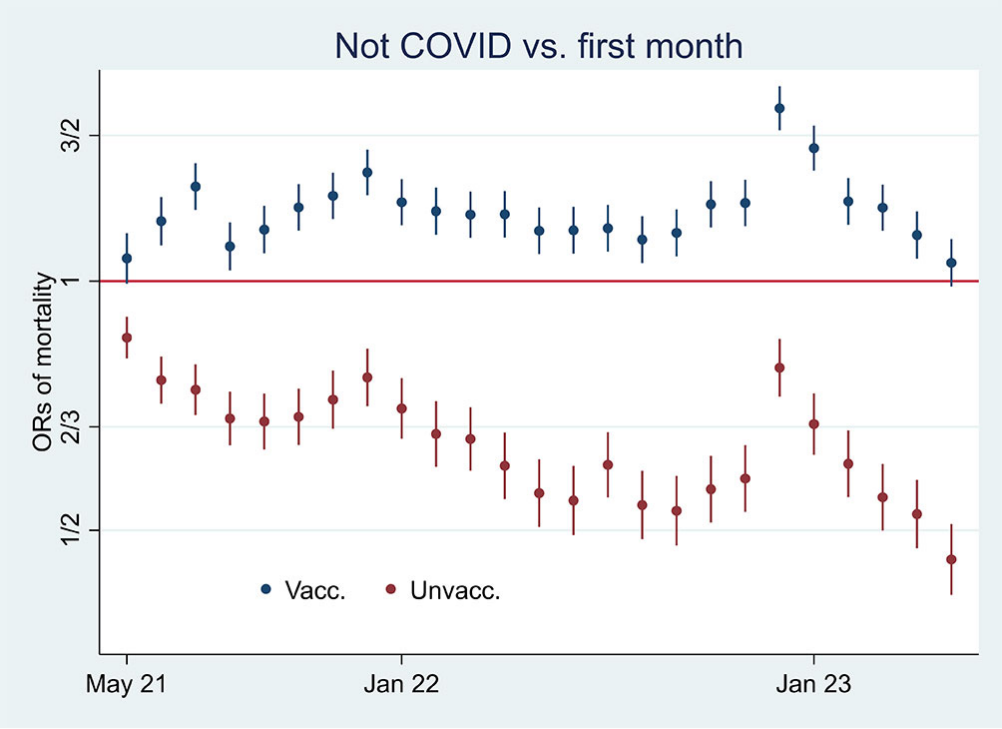
Monthly ORs of mortality with 95% CIs.

**Figure 4.**
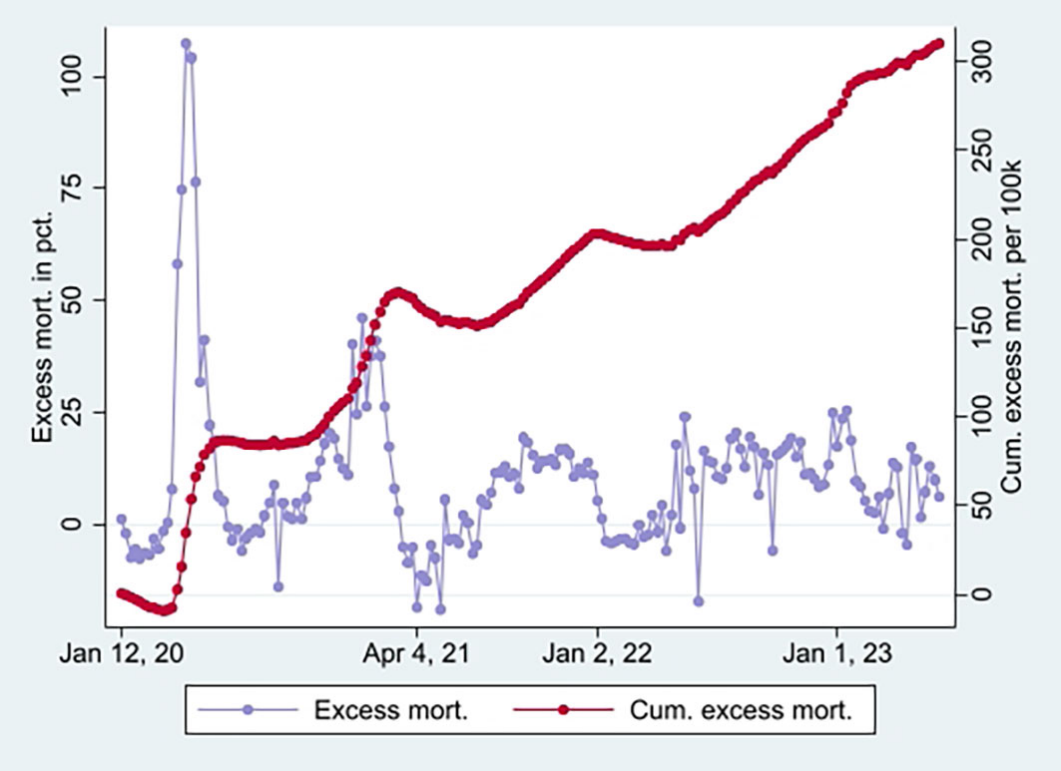
Weekly UK excess mortality in percent and cumulative excess mortality.

Figure 3 shows that while mortality not involving COVID-19 decreased among unvaccinated compared to the first observation month, it was high among vaccinated [ 4]. The results reflect mortality rates in Figure 1B, which were almost tangent at the end of the period. Also, they reflect the declining ORs of unvaccinated reported in Figure 2A, taking a non-significant value of a little over 1 at the end (95% CI). Hence, the data show a relatively high and relative increase in mortality not involving COVID-19 among vaccinated. An interpretation is that vaccination, despite temporary protection, increased mortality. Strengthening the interpretation was relatively high mortality among vaccinated not involving COVID-19 counterintuitively following periods of excess mortality ( Figure 4) [ 5]. Further strengthening the interpretation was the relatively high mortality not involving COVID-19 among the vaccinated, corresponding with excess mortality during much of the same period (ibid.) [ 6].

**Figure 5.**
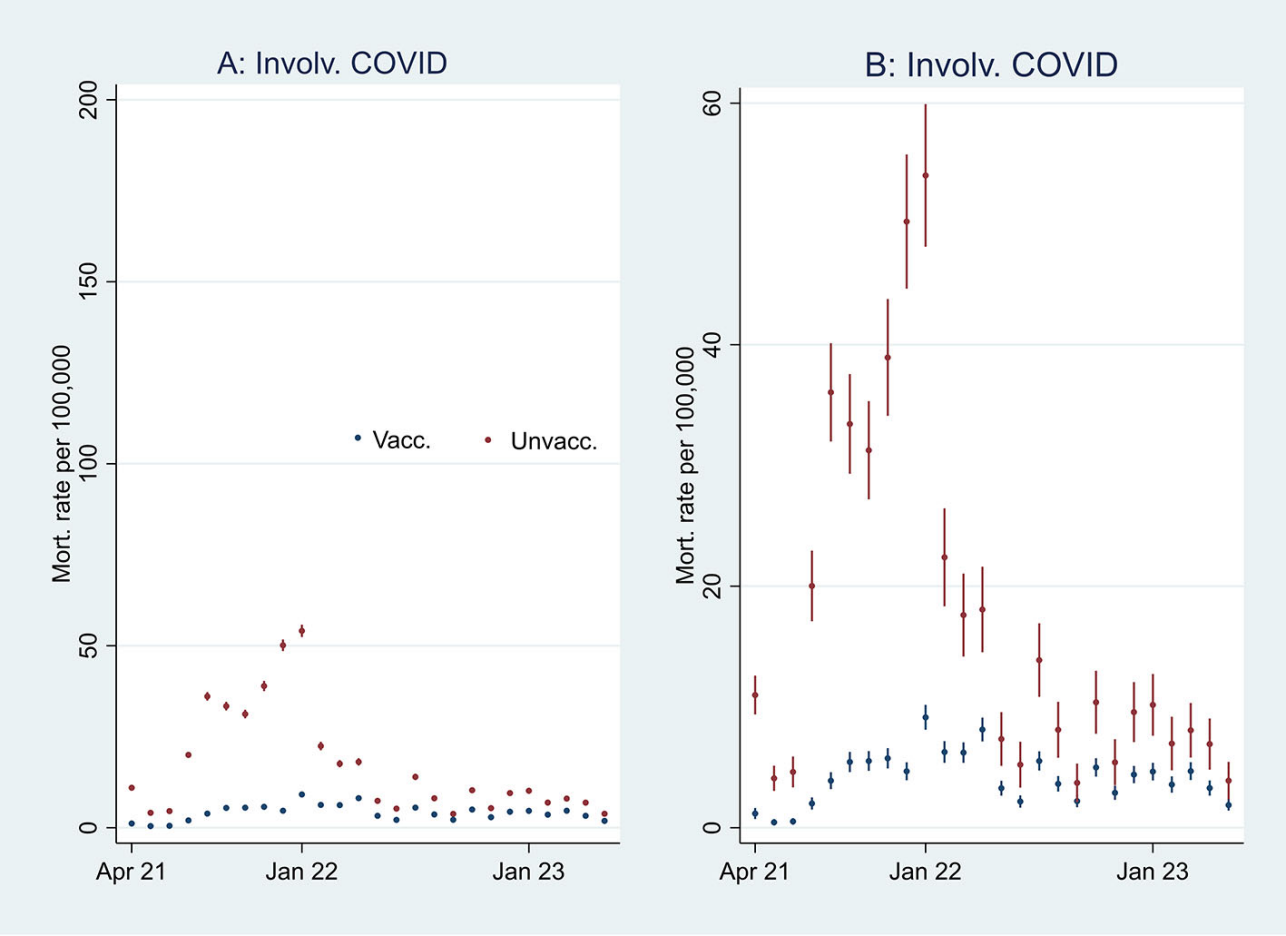
Monthly mortality rates involving COVID-19 with 95% CIs.

**Figure 6.**
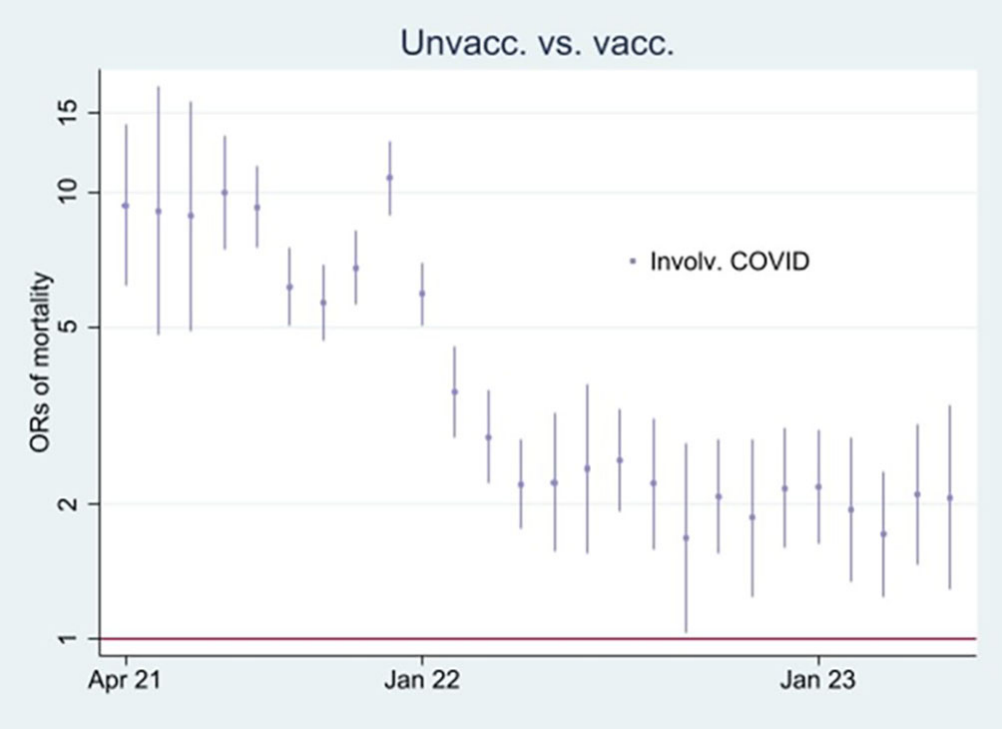
Monthly ORs of mortality involving COVID-19 with 95% CIs.

## Discussion

This study found that COVID-19 vaccination protected against mortality, but the effect was temporal and declined after a few months. Also, the study found that COVID-19 vaccination may have increased mortality in a longer perspective.

As the study found that COVID-19 vaccination prevented mortality, it contributes to and aligns with other research showing similar effects. ^11^^–^^17^ As it found that the vaccine protection was temporal, it further contributes to and aligns with other research showing that it declines. ^19^ Finally, as the study found that COVID-19 may have increased mortality in a longer perspective, it contributes to and aligns with other research also showing that COVID-19 vaccination can have adverse effects ^27– 29^ and increase mortality. ^30^

In addition to contributing to the other research streams concerning the COVID-19 vaccine effect on mortality, the study’s perhaps major contribution was to elaborate a useful tool to compare non-randomized groups in the absence of control variables, which even in their presence can even make statistical conclusions less, not more, accurate. ^6^ Thus, as most previous studies on the link between COVID-19 vaccination and mortality have been carried out in non-randomized contexts and, accordingly, even in the presence of control variables exposed to challenges concerning validity, this study has illustrated and applied a useful tool to address those limitations. Moreover, I argue that the tool has general applicability as it can also be used in other research contexts.

### Implications

Predicting outcomes of future potential pandemics is challenging, ^31^ highlighting the importance of high-quality healthcare sectors as they have been shown to prevent adverse outcomes. ^32^ Lessons from the COVID-19 pandemic have nonetheless taught that the “proportion of adults hospitalized with COVID-19 who experienced critical outcomes decreased with time”, ^33^ but the statement does not undermine its challenge on the society at large and the health care sector in particular. This study has shown that vaccination, although having a temporal preventive effect, can even have adverse long-term consequences. Policymakers and the healthcare sector should be aware of these findings, considering that the effect of the COVID-19 vaccine is not necessarily genuinely positive.

### Limitations and future research

During the study period, a share of people in the unvaccinated group were transferred to the vaccinated. Assuming they had inferior health status at the outset, it may explain the relative increase (decrease) in mortality among the vaccinated (unvaccinated). However, those who *remained* unvaccinated, on the contrary, had inferior health status at the outset, ^1^ making the above reasoning implausible. Ceteris paribus, one may even oppositely conclude that it would decrease (increase) relative mortality among vaccinated (unvaccinated) [ 7]. Since most elderly candidates had been offered vaccine before Apr 21, ^1, 34^ I nonetheless assume the estimates were not substantially skewed over the study period, as relatively few people die in younger age cohorts.

The study’s validity hinges on non-systematic skewness in classifying false positives concerning mortality involving COVID-19 and false negatives concerning mortality not involving COVID-19. However, I cannot see any substantial reason for substantial skewness in false positives and negatives between vaccinated and unvaccinated, but it may induce some cautiousness when interpreting the data.

The validity of the finding that vaccinated had significant protection between July 21 and Jan 22 hinges on non-systematic skewness in classifying false positives concerning mortality involving COVID-19 and false negatives concerning mortality not involving COVID-19. A relevant issue in this regard is that the English data excluded ICD10 death certificate codes U09.9 (Post-COVID condition, where the acute COVID had ended before the condition immediately causing death occurred) and U10.9 (Multisystem inflammatory syndrome associated with COVID-19) as criteria when classifying mortality involving COVID-19, but as this was the case only when the U07.1 (COVID-19, virus identified) or U07.2 (COVID-19, virus not identified) were *not* mentioned, I cannot see substantial skewness in false positives and negatives between vaccinated and unvaccinated. The potential limitation may nonetheless induce cautiousness when interpreting the data, which I encourage future research to address.

The validity of the finding that vaccinated had non-significant protection from Feb 22 also has limitations, as relatively low mortality involving COVID-19 can be an alternative explanation. However, in Note [3], I elaborate extensively on the issue, concluding that the alternative explanation is not very likely, but I nonetheless encourage cautiousness when interpreting the data.

This study included those ten years and older. I, therefore, encourage future research to analyze different age cohorts separately to assess how findings may converge or eventually diverge. As this study merely distinguished between those vaccinated and those who were not, I also encourage future research to distinguish between those who received one or more doses and different vaccine types, although it may be methodologically challenging.

### Ethics and consent

Ethical approval and consent were not required.

## Data Availability

UK Office for National Statistics. ^9^ Deaths by vaccination status, England 2023: https://www.ons.gov.uk/peoplepopulationandcommunity/birthsdeathsandmarriages/deaths/datasets/deathsbyvaccinationstatusengland I used the dataset labeled “Deaths occurring between 1 April 2021 and 31 May 2023 edition of this dataset”, Table 1: Unvaccinated and Ever vaccinated. The Methods section explains in detail how I modeled the data.

## Footnotes

^1^
Vertical axes in Figure 2 are log-transformed using the natural logarithm.

^2^
Overlapping 95% CIs July 21 in Figure 2A appears inconsistent with significant OR (95% CI) for the same month in Figure 2B, but the issue is discussed by Knol, Pestman and Grobbee. ^35^

^3^
One may attribute the seemingly non-significant vaccine protection from Feb 22 ( Figure 2B) to relatively low mortality involving COVID-19 during that period ( Figure 5 below shows the data where A and B are identical, except for different scaling). The reason for the argument is that the effects in Figure 2B would be absent if the mortality involving COVID-19 approached zero (which explains the non-significant effect between Apr and Jun 21). However, among the vaccinated, the mortality rate in several months from Feb 22 was higher than in months between Jul 21 and Jan 22, countering that argument. Moreover, Figure 6 below shows that the ORs of mortality among unvaccinated (compared to vaccinated as a reference group) involving COVID-19 were down from about 10 at the beginning to about 2 at the end. It implies that not only was the mortality among unvaccinated not involving COVID-19 relatively low at the end ( Figure 2A), but also the mortality involving COVID-19 ( Figure 6). Taken together, the decrease in mortality involving COVID-19 largely occurred among the unvaccinated. The ORs in Figure 6 were significant (95% CIs) during the whole period, which can be due to (i) vaccine protection and (ii) unvaccinated having inferior health at the outset. ^1^ However, as the ORs were only about a fifth since Feb 22 compared to the first months, explanation (ii) is more likely during that period.

^4^
The overall pattern among unvaccinated was similar both concerning all-cause mortality and mortality not involving COVID-19. Therefore, one cannot claim that the overall decrease in mortality not involving COVID-19 was due to mortality involving it.

^5^
Assuming that the excess mortality among the unvaccinated segment before Apr 21 was *a* percent, taking a positive value, one may assume that it was *a** *b* percent among the vaccinated segment, where 0< *b*<1. One may assume *b*<1 because the vaccinated segment had relatively good health at the outset, ^1^ and one may assume 0< *b* because there were, nonetheless, people vulnerable to COVID-19 among them. I.e., *a** *b* was lower than *a* but still higher than zero. According to the reasoning, one should expect a decline in mortality among vaccinated during the study period due to previous excess mortality, but not necessarily as marked as observed among unvaccinated. Alternatively, one may argue the opposite as among the vaccinated segment, “some very comorbid patients [in care homes] got vaccine side effects that probably accelerated an already progressing death process” (Ref. 36, p. 3 - my translation from Norwegian).

^6^

Figure 4 shows weekly UK excess mortality in percent and cumulative excess mortality. ^37^ English monthly data ^38^ show similar patterns concerning excess mortality in percent. For an extensive review of all-cause mortality in England and Wales, please see Jones and Ponomarenko (2023). ^39^

^7^
People in England under 70 years old but clinically extremely vulnerable were prioritized vaccination with those aged between 70-74. ^34^ Hence, they were prioritized early.
