# Mortality involving and not involving COVID-19 among vaccinated vs. unvaccinated in England between Apr 21 and May 23

**DOI:** 10.12688/f1000research.160980.5

**Published:** 2026-02-14

**Authors:** Jarle Aarstad

**Affiliations:** 1 Western Norway University of Applied Sciences, Bergen, Norway

**Keywords:** COVID-19 vaccination; all-cause mortality; mortality involving COVID-19; mortality not involving COVID-19; excess mortality.

## Abstract

**Background:**

Comparing non-randomized groups, such as COVID-19 vaccinated and unvaccinated, even in the presence of seemingly relevant control variables, is challenging, but in this study, using English data, I show an achievable approach.

**Methods:**

First, I estimated age-standardized all-cause mortality among vaccinated and unvaccinated ten years and older, covering 26 months from Apr 21 to May 23. Then, I estimated mortality not involving COVID-19, and finally, I contrasted the calculations.

**Results:**

First, I found that all-cause mortality among unvaccinated was higher than among vaccinated. But, as the pattern was similar concerning mortality not involving COVID-19, the discrepancy can be attributed mainly to unvaccinated having inferior health at the outset. There were nonetheless indications of significant protection for vaccinated between July 21 and Jan 22. In the absence of control variables as a means to compare non-randomized groups, I reached that conclusion by contrasting all-cause mortality with mortality not involving COVID-19. However, while mortality not involving COVID-19 decreased among unvaccinated compared to the first observation month, it remained high among vaccinated, indicating a relative increase.

**Conclusions:**

An interpretation is that vaccination, despite a potential temporary protection, has increased mortality. Strengthening the interpretation was relatively high mortality among vaccinated not involving COVID-19 counterintuitively following periods of excess mortality. Further strengthening the interpretation was the relatively high mortality not involving COVID-19 among vaccinated, corresponding with excess mortality during much of the same period. Future research should include data over a longer period than those available for this study. Also, future research should examine different age groups, vaccination types, and the number of doses given.

## Introduction

According to the UK Office for National Statistics, ^1^ rates for COVID-19 unvaccinated adults in England “were higher for Black Caribbean, Black African and White Other ethnic groups. Rates were also higher for those living in deprived areas, who have never worked or are long-term unemployed, who are limited a lot by a disability, … or who are male.” The statement aligns with vaccine hesitancy research ^2, 3^ and further indicates that unvaccinated have inferior health at the outset compared to vaccinated, inducing biased comparisons as the groups are not randomly assigned. Therefore, matching, balancing, ^4^ or controlling for potential confounders, e.g., ethnicity, employment-, disability-, socioeconomic status, and gender, can debias the results. ^5^ However, variables accounting for potentially confounding effects are often unavailable or unknown, and including those available but unknowingly improper can increase bias. ^6^ In line with the reasoning, York (Ref. 6, p. 675) showed that “unless *all* potential confounding factors are included in an analysis (which is unlikely to be achievable with most real-world data-sets), adding control variables to a model in many circumstances can make estimated effects … less accurate.” Norwegian research exemplifies that showing 30% lower all-cause mortality among COVID-vaccinated compared unvaccinated, 18-44 years, and 58% when including control variables. ^7^ The findings are unattributable to a vaccine effect as close to zero young people died of COVID-19 in Norway, ^8^ and illuminate two issues: (i) COVID-19 vaccinated and unvaccinated have different health status at the outset and (ii) including control variables can make estimates less, not more, accurate, both consistent with my outline above.

Hence, I argue there is a research gap concerning valid estimations between non-randomized groups, such as COVID-19 vaccinated and unvaccinated, which is challenging even when including seemingly relevant control variables that can actually deteriorate the results. ^7^ To address the research gap, using English data covering 26 months from Apr 21 to May 23, ^9^ I explain an achievable approach by contrasting all-cause mortality among COVID-19 vaccinated and unvaccinated with mortality not involving COVID-19 among COVID-19 vaccinated and unvaccinated.

The study’s research question is accordingly as follows: Applying the approach addressed above, how do the mortality patterns differ in England from Apr 21 to May 23 between COVID-19 vaccinated and unvaccinated? The study’s major contribution is to illustrate how contrasting all-cause mortality with mortality not involving COVID-19 can indicate valid estimates between non-randomized groups of vaccinated and unvaccinated.

Studies have indicated that COVID-19 vaccination can prevent mortality, ^10– 16^ but the effect declines. ^17^ Applying my approach to the English data, I particularly contribute to the research on the link between COVID-19 vaccination and mortality, as most previous studies have been carried out in non-randomized contexts and, accordingly, even in the presence of control variables, exposed to challenges concerning validity addressed above.

## Methods

### Sample and data

I used publicly available data on the population in England aged ten years and older provided by the UK Office for National Statistics, ^9^ for this study. Particularly, I applied their data on monthly age-standardized all-cause mortality and mortality not involving COVID-19 by vaccination status, ^18, 19^ and present further details below. The period for which data were available and included in this study was between Apr 21 and May 23, 26 months.

### Measures of variables

The study includes the two effect variables, monthly mortality rates and monthly odds ratios (ORs) of mortality. As noted, I distinguished between all-cause mortality and mortality not involving COVID-19. All-cause mortality implies anybody who died regardless of cause. Mortality not involving COVID-19 implies those who died but did *not* have COVID-19 mentioned on the death certificate in terms of ICD10 codes U07.1 (COVID-19, virus identified) or U07.2 (COVID-19, virus not identified).

COVID-19 vaccinated for this study were those having received one or more doses, labeled as “ever vaccinated” in the raw data, and unvaccinated were those not having received any dose. Each month, I classified those who either died of any cause (all-cause mortality) or survived as either COVID-19-vaccinated or unvaccinated. Hence, each month, a person in the data was classified as (i) dead and vaccinated, (ii) alive and vaccinated, (iii) dead and unvaccinated, or (iv) alive and unvaccinated. I made similar classifications concerning mortality not involving COVID-19.

To exemplify, in Apr 21, the age-standardized all-cause mortality rate among “ever vaccinated”, i.e., defined as vaccinated in this study, was 812.7 per 100,000 person-years, which were 2,124,523 that month. ^9^ The expression (812.7/100,000)*2,124,523 gives 17,266 estimated deaths in an estimated population of 25,494,276, which was reached by multiplying 2,124,523 by 12. I.e., the age-standardized all-cause mortality rate per 100,000 vaccinated in Apr 21 was 17,266 divided by 25,494,276 multiplied by 100,000, resulting in a value of 67.7. Similar estimations of all-cause mortality and mortality not involving COVID-19, were carried out each month for vaccinated and unvaccinated. (Also, I present mortality estimations *involving* COVID-19. I.e., estimations excluding mortality not involving COVID-19.) I conduct the exercise, assessing how many died or survived in a population during a given month, whether vaccinated or unvaccinated, to estimate as statistically correct standard errors as possible using logistic regression.

### Models and data analysis procedure

The data were applied in logistic regressions using Stata 17. ^20^ I used the margin effect command to estimate mortality rates, ^21^ followed by OR estimations.

Initially, I (i) estimated monthly age-standardized all-cause mortality rate per 100,000 among COVID-19 vaccinated and unvaccinated. Then, I (ii) estimated mortality rate not involving COVID-19, and finally, using xlincom, ^22^ an extension of Stata’s ^20^ lincom algorithm, I contrasted the results between (i) and (ii), and presented them as ORs. Concerning ORs, I particularly explain and show in the Results section how the xlincom algorithm was used to contrast log odds (the logarithm of the ORs) estimates. Also, I explain the substantial interpretation of contrasted estimates.

As all-cause mortality estimates include cases involving COVID-19, I will argue that contrasting those with estimates not involving COVID-19 cases can illuminate vaccination effects between populations with potentially different health statuses at the outset.

## Results

**Figure 1.**
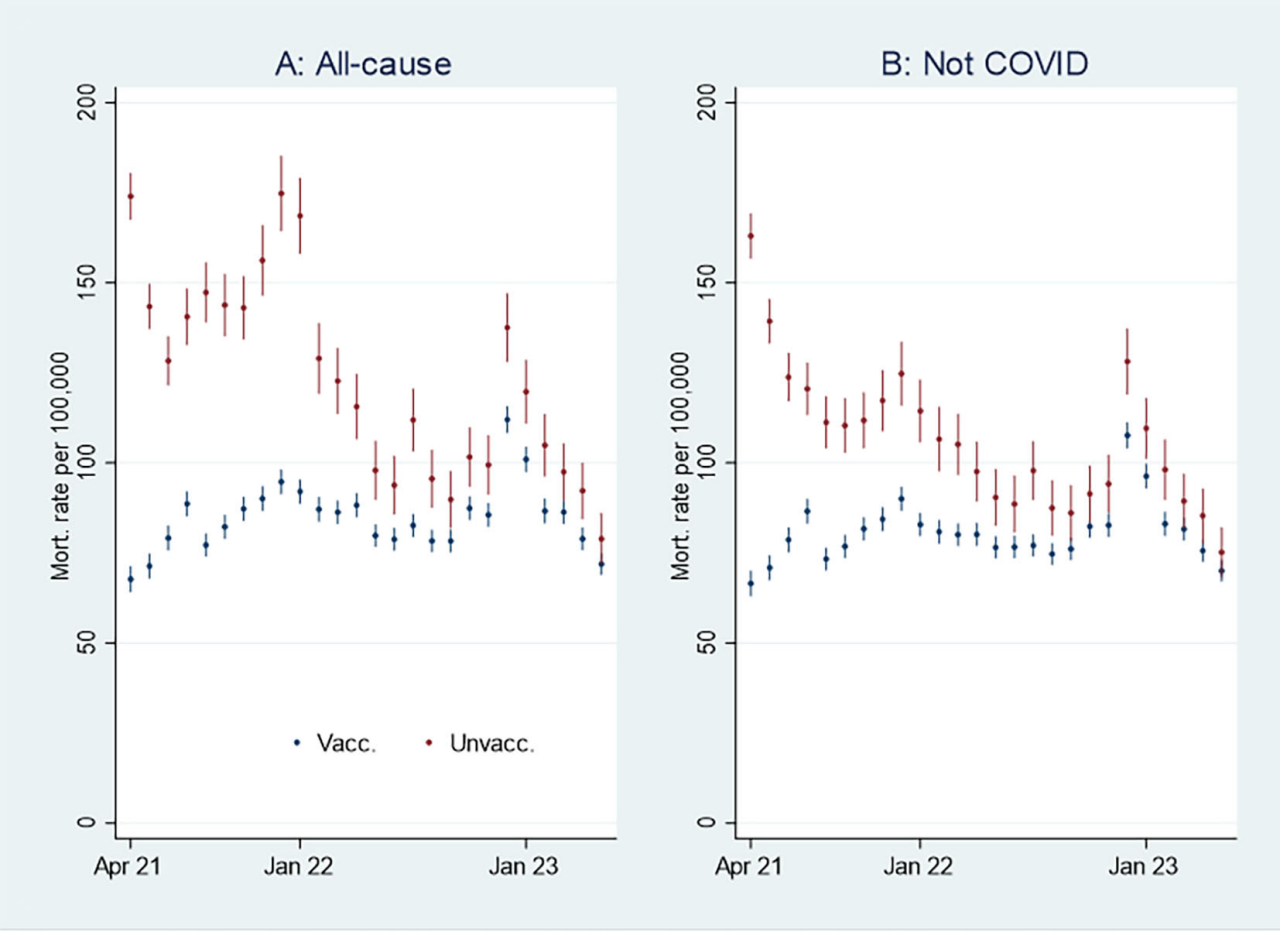
Monthly mortality rates per 100,000 with 95% CIs.

**Figure 2.**
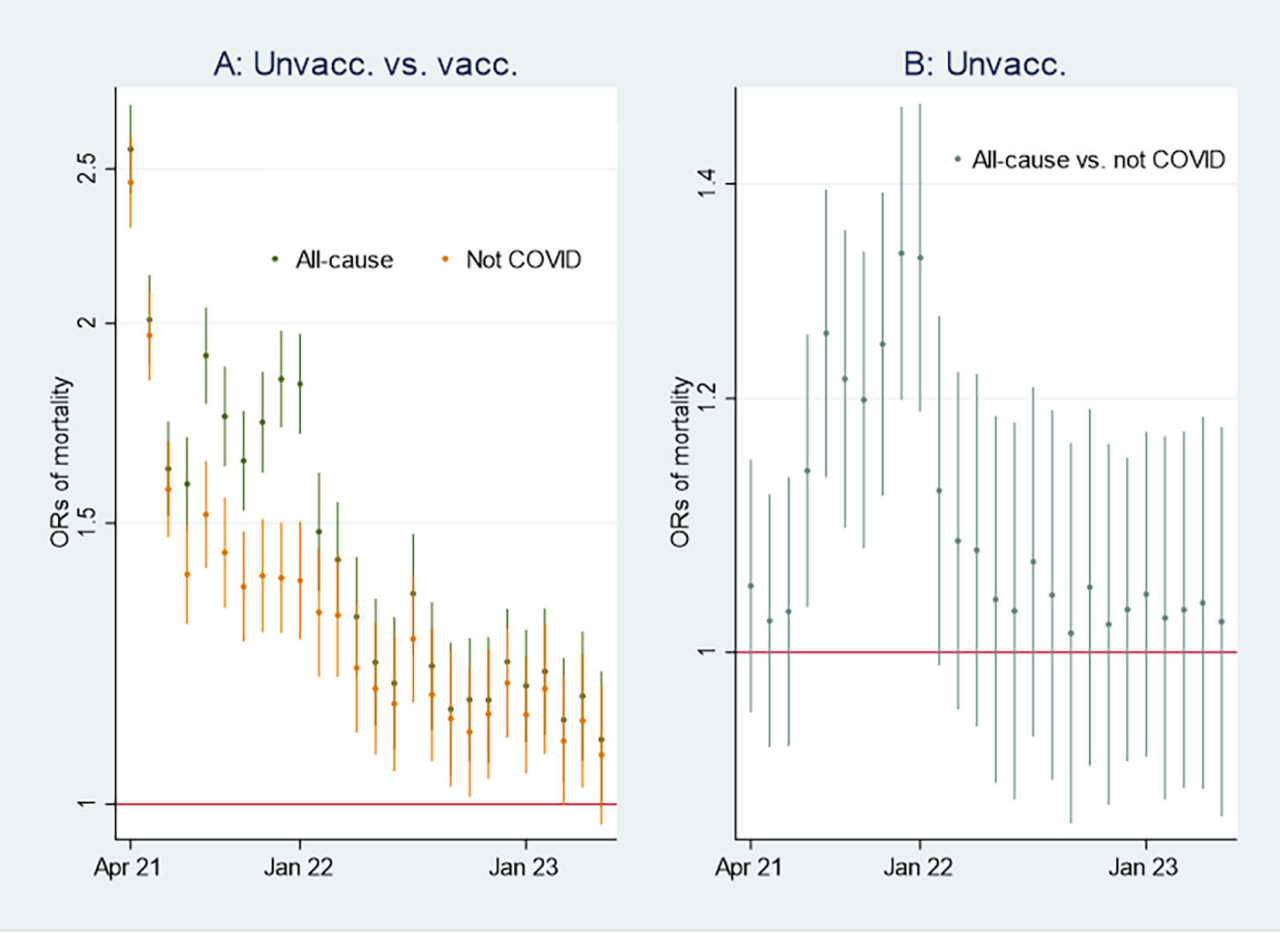
Monthly ORs of mortality with 95% CIs.

**Figure 3.**
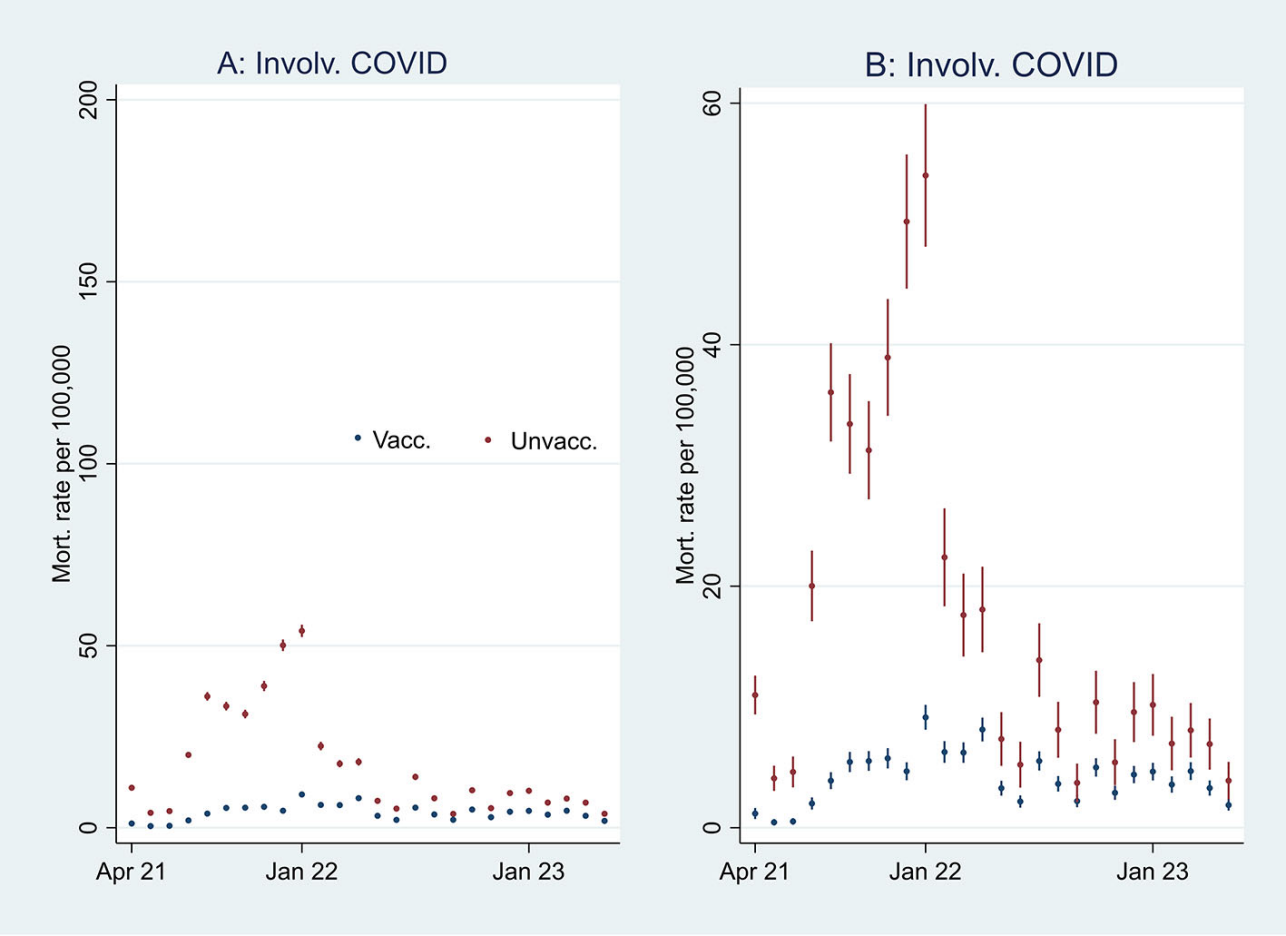
Monthly mortality rates involving COVID-19 with 95% CIs.

**Figure 4.**
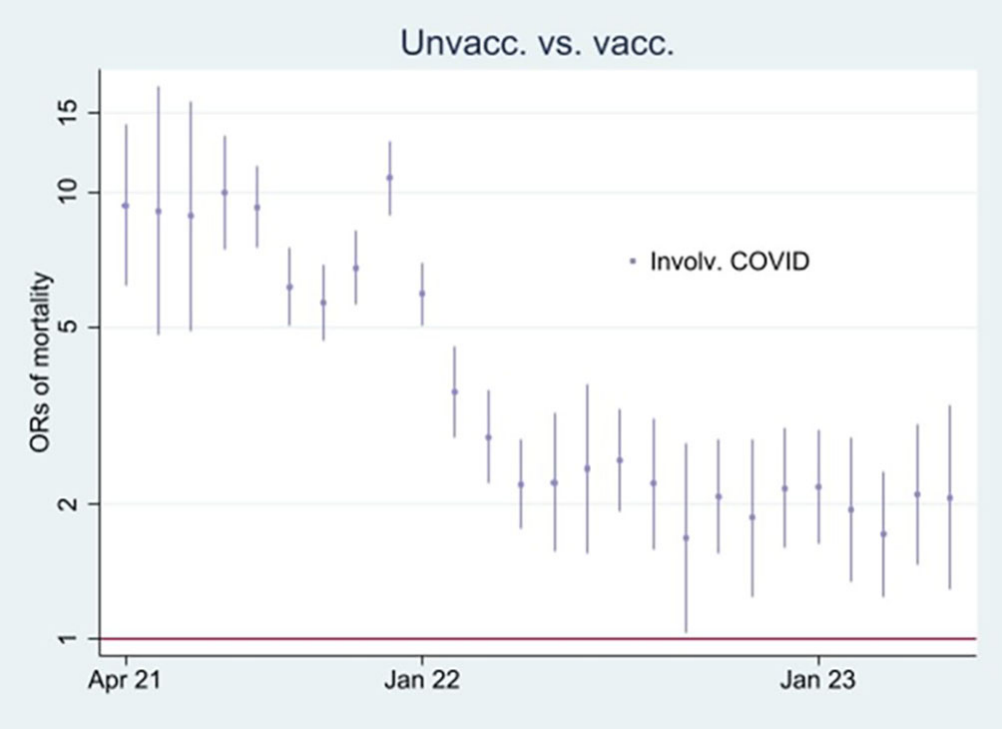
Monthly ORs of mortality involving COVID-19 with 95% CIs.

I first present the empirical results of age-standardized mortality rates among vaccinated and unvaccinated aged ten years and older, shown in Figure 1. Aided by odds ratios (ORs) calculations shown in Figure 2, I then address the results’ substantial interpretation. To do so, I also present mortality rates and ORs involving COVID-19 in Figures 3 and 4, respectively.

### Initial mortality rate analyses

Figure 1A shows that the monthly all-cause mortality rate, particularly at the beginning of the period, was higher among unvaccinated (marked in red) than vaccinated (marked in blue). The rate decreased among the unvaccinated, but among the vaccinated, it was relatively stable or had a slight increase. Consequently, the all-cause mortality among unvaccinated and vaccinated was almost tangent at the end of the period.

Figure 1B shows that the mortality rate not involving COVID-19 was similar to the all-cause mortality rate ( Figure 1A), except for being lower among unvaccinated between the last half of 21 and the beginning of 22.

An interpretation of Figure 1A can be that the vaccinated had a temporal but declining mortality protection. However, as the pattern was similar concerning mortality not involving COVID-19 ( Figure 1B), there may be other explanations, which I address below.

### Odds ratio analyses and mortality involving COVID-19

Figure 2A shows ORs of all-cause mortality and mortality not involving COVID-19 among unvaccinated compared to vaccinated as a reference group [ 1]. At the beginning of the period, the ORs of all-cause mortality (marked in green) among unvaccinated were approximately between 2 and 2.5 compared to vaccinated (significant at the 95% CIs), and mortality not involving COVID-19 (marked in orange) shows a similar pattern. In parallel, Figure 3 shows that the mortality rate involving COVID-19 was low at the beginning of the period for both vaccinated and unvaccinated (A and B are identical, except for different scaling). Therefore, I conclude that unvaccinated had between 2 and 2.5 times higher ORs of all-cause mortality and mortality not involving COVID-19 compared to vaccinated at the beginning of the period, largely due to inferior health at the outset, and not vaccine protection since the overall mortality involving COVID-19 during that period was low. The argument is grounded in the assumption that the vaccine unlikely protects against mortality not involving COVID-19. ^23^ That is, if close to zero people died from COVID-19, I cannot see any logical reason why the mortality pattern observed at the beginning of the period has another explanation than unvaccinated having inferior health at the outset.

Between the last half of 21 and the beginning of 22, on the other hand, the ORs were higher for all-cause mortality than for mortality not involving COVID-19 ( Figure 2A), which may indicate a temporal preventive vaccine effect. Figure 3 supports that assumption as it particularly shows an uptick in the mortality rate involving COVID-19 among unvaccinated during that period.

Figure 2B shows that ORs of all-cause mortality compared to mortality not involving COVID-19 between July 21 and Jan 22 were significant (95% CIs), with most values above 1.2. The results were reached by using xlincom, ^22^ an extension of Stata’s ^20^ lincom algorithm, first to contrast or differentiate the log odds (the logarithm of the ORs) of estimates reported in Figure 2A, and next to generate new ORs (from the contrasted or differentiated log odds). Substantially, Figure 2A and Figure 2B provide the same information [ 2], but in my opinion, the latter illuminates the contrast between all-cause mortality and mortality not involving COVID-19 better [ 3] explains Figure 4.

### What odds ratios and mortality rates may indicate over time

**Figure 5.**
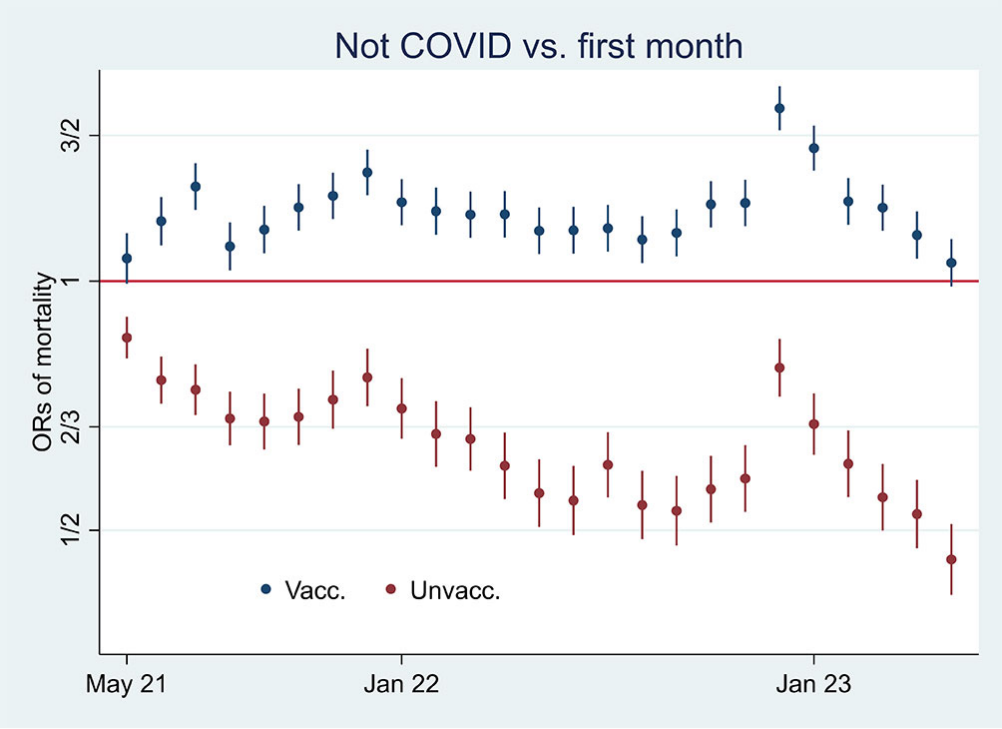
Monthly ORs of mortality with 95% CIs.

**Figure 6.**
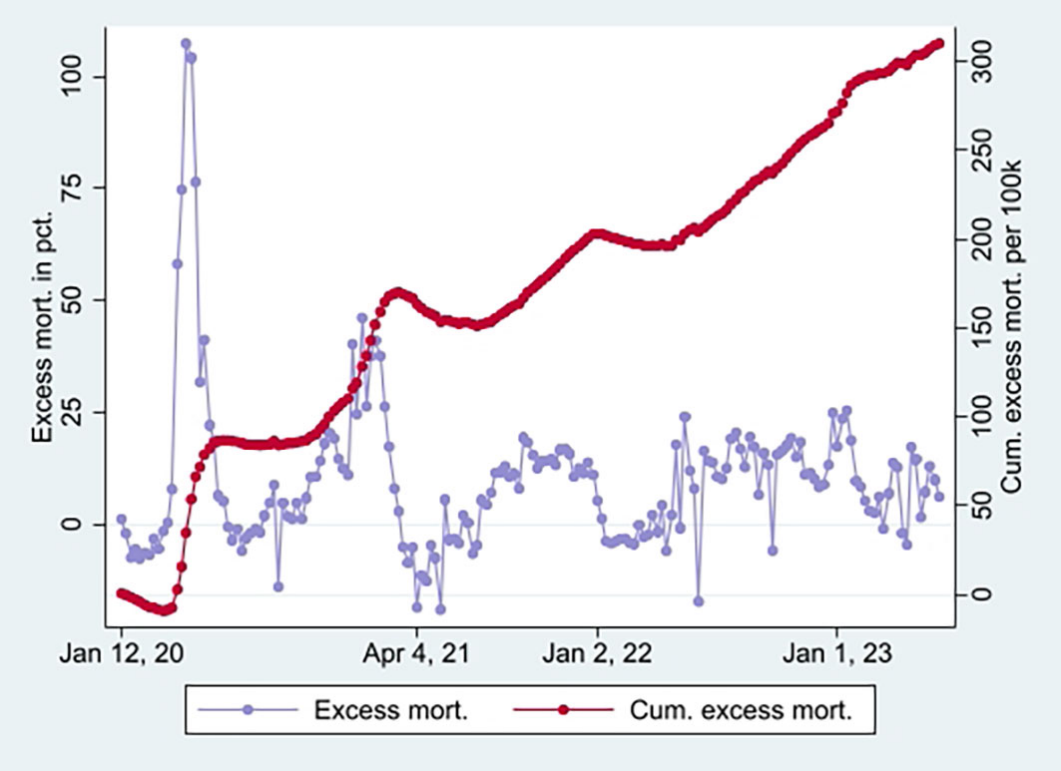
Weekly UK excess mortality in percent and cumulative excess mortality.

Figure 5 shows that while mortality not involving COVID-19 decreased among unvaccinated (marked in red) compared to the first observation month, it remained high among vaccinated (marked in blue) [ 4]. The results reflect mortality rates in Figure 1B, which were almost tangent at the end of the period. Also, they reflect the declining ORs of unvaccinated reported in Figure 2A (marked in orange), taking a non-significant value of a little over 1 at the end (95% CI). Hence, the data show a relatively high and relative increase in mortality not involving COVID-19 among vaccinated. An interpretation is that vaccination, despite temporary protection, increased mortality. Strengthening the interpretation was relatively high mortality among vaccinated not involving COVID-19 counterintuitively following periods of excess mortality ( Figure 6) [ 5]. Further strengthening the interpretation was the relatively high mortality not involving COVID-19 among the vaccinated, corresponding with excess mortality during much of the same period (ibid.) [ 6].

## Discussion

This study found that COVID-19 vaccination may have protected against mortality, but the effect was temporal and declined after a few months. Also, the study indicated that COVID-19 vaccination can have increased mortality in the long term.

As the study found that COVID-19 vaccination may have prevented mortality, it contributes to and aligns with other research showing similar effects. ^10^^–^^16^ As it found that the vaccine protection was temporal, it further contributes to and aligns with other research showing that it declines. ^17^ Finally, as the study indicated that COVID-19 vaccination may have increased mortality in a longer perspective, it contributes to and aligns with other research that also shows the intervention can have adverse effects ^24– 27^ and increase mortality, ^28^ including from the virus. ^29^^,^^30^

In addition to contributing to the other research streams concerning the COVID-19 vaccine effect on mortality, the study’s perhaps major contribution was to elaborate a useful tool to compare non-randomized groups in the absence of control variables, which, even in their presence, can make statistical conclusions less, not more, accurate. ^6^ Thus, as most previous studies on the link between COVID-19 vaccination and mortality have been carried out in non-randomized contexts and, accordingly, even in the presence of control variables exposed to validity concerns, this study has illustrated and applied a useful tool to address those limitations. Moreover, I argue that the tool has general applicability as it can also be used in other research contexts.

### Implications

Predicting outcomes of future potential pandemics is challenging, ^31^ highlighting the importance of high-quality healthcare sectors as they have been shown to prevent adverse outcomes. ^32^ Lessons from the COVID-19 pandemic have nonetheless taught that the “proportion of adults hospitalized with COVID-19 who experienced critical outcomes decreased with time”, ^33^ but the statement does not undermine its challenge on society at large and the health care sector in particular. This study has shown that vaccination, although having a temporal preventive effect, can have adverse long-term consequences. Policymakers and the healthcare sector should be aware of these findings, considering that the effect of the COVID-19 vaccine is not necessarily genuinely positive.

### Limitations and future research

During the study period, a share of people in the unvaccinated group were transferred to the vaccinated. Assuming they had an inferior health status at the outset, it may explain the relative increase (decrease) in mortality among the vaccinated (unvaccinated). However, those who *remained* unvaccinated, on the contrary, had inferior health status at the outset, ^1^ making the above reasoning implausible. Ceteris paribus, one may even oppositely conclude that it would decrease (increase) relative mortality among vaccinated (unvaccinated) [ 7]. Since most elderly candidates had been offered the vaccine before Apr 21, ^1, 34^ I nonetheless assume the estimates were not substantially skewed over the study period, as relatively few people die in younger age cohorts.

The validity of the finding indicating that vaccinated had significant protection between July 21 and Jan 22 hinges on non-systematic skewness in classifying false positives concerning mortality involving COVID-19 and false negatives concerning mortality not involving COVID-19. A relevant issue in this regard is that the English data excluded ICD10 death certificate codes U09.9 (Post-COVID condition, where the acute COVID had ended before the condition immediately causing death occurred) and U10.9 (Multisystem inflammatory syndrome associated with COVID-19) as criteria when classifying mortality involving COVID-19, but as this was the case only when the U07.1 (COVID-19, virus identified) or U07.2 (COVID-19, virus not identified) were *not* mentioned, I cannot see substantial skewness in false positives and negatives between vaccinated and unvaccinated. The potential limitation may nonetheless induce cautiousness when interpreting the data, which I encourage future research to address.

The validity of the finding that vaccinated had non-significant protection from Feb 22 also has limitations, as relatively low mortality involving COVID-19 can be an alternative explanation. However, in Note [ 3], I elaborate on the issue, concluding that the alternative explanation is not very likely, but I nonetheless encourage cautiousness when interpreting the data.

This study included those ten years and older. I, therefore, encourage future research to analyze different age cohorts separately to assess how findings may converge or eventually diverge. As this study merely distinguished between those vaccinated and those who were not, I also encourage future research to distinguish between those who received one or more doses and different vaccine types, although it may be methodologically challenging.

### Ethics and consent

Ethical approval and consent were not required.

## Data Availability

UK Office for National Statistics. ^9^ Deaths by vaccination status, England 2023: https://www.ons.gov.uk/peoplepopulationandcommunity/birthsdeathsandmarriages/deaths/datasets/deathsbyvaccinationstatusengland I used the dataset labeled “Deaths occurring between 1 April 2021 and 31 May 2023 edition of this dataset”, Table 1: Unvaccinated and Ever vaccinated. The Methods section explains in detail how I modeled the data.

## Footnotes

^1^
Vertical axes in Figure 2 are log-transformed using the natural logarithm.

^2^
Overlapping 95% CIs for July 21 in Figure 2A appears inconsistent with a significant OR (95% CI) for the same month in Figure 2B, but Knol, Pestman, and Grobbee discuss the issue. ^35^

^3^
One may attribute the non-significant effect from Feb 22 ( Figure 2B) to relatively low mortality involving COVID-19 from that month ( Figure 3). The reason for the argument is that the effects in Figure 2B would be absent if the mortality involving COVID-19 approached zero (which explains the non-significant effect between Apr and Jun 21). It is nonetheless worth noting, that among the vaccinated, the mortality rate in several months from Feb 22 was higher than in months between Jul 21 and Jan 22 ( Figure 3). Hence, the decrease in mortality involving COVID-19 largely occurred among the unvaccinated. This is reflected in ORs of mortality involving COVID-19 among unvaccinated (compared to vaccinated as a reference group), decreasing from about 10 at the beginning to about 2 at the end ( Figure 4). One may further attribute the non-significant effect from Feb 22 ( Figure 2B) to population immunity, as the majority of the population was vaccinated, protecting the unvaccinated, a form of indirect protection. However, from early 2022, Omicron became the dominant variant, for which vaccination provided limited indirect protection after three months. ^36^

^4^
The overall pattern among unvaccinated was similar both concerning all-cause mortality and mortality not involving COVID-19. Therefore, one cannot claim that the overall decrease in mortality not involving COVID-19 was due to mortality involving it.

^5^
Assuming that the excess mortality among the unvaccinated segment (those that would not opt for vaccination) before Apr 21 was *a* percent, taking a positive value, one may assume that it was *a** *b* percent among the vaccinated segment (those that would opt for vaccination), where 0< *b*<1. One may assume *b*<1 because the vaccinated segment had relatively good health at the outset, ^1^ and one may assume 0< *b* because there were, nonetheless, people vulnerable to COVID-19 among them. I.e., *a** *b* was lower than *a* but still higher than zero. According to the reasoning, one should expect a decline in mortality among vaccinated during the study period due to previous excess mortality, but not necessarily as marked as observed among unvaccinated. A parallel argument is that the excess mortality before Apr 21 cannot be attributed to the unvaccinated segment alone, as they only represent roughly 10% of the English population, ten years and older. Alternatively, one may argue the opposite as among the vaccinated segment, “some very comorbid patients [in care homes] got vaccine side effects that probably accelerated an already progressing death process” (Ref. 37, p. 3 - my translation from Norwegian).

^6^

Figure 6 shows weekly UK excess mortality in percent and cumulative excess mortality. ^38^ English monthly data ^39^ show similar patterns concerning excess mortality in percent. For an extensive review of all-cause mortality in England and Wales, please see Jones and Ponomarenko (2023). ^40^

^7^
People in England under 70 years old but clinically extremely vulnerable were prioritized for vaccination, with those aged between 70 and 74. ^34^ Hence, they were prioritized early.
