# Supplementary material for: Mortality involving and not involving COVID-19 among vaccinated vs. unvaccinated in England between Apr 21 and May 23
Source: F1000Res. 2026 Feb 14;14:133. Originally published 2025 Jan 27. [Version 5] doi: 10.12688/f1000research.160980.5 (PMC13033977; doi:10.12688/f1000research.160980.5)
Supplement: Peer review history

# Reviewer report: Reviewer response for version 5

**DOI:** 10.5256/f1000research.196436.r459362

**Published:** 2026-03-29

**Authors:** Cecilia Acuti Martellucci

**Affiliations:** 1 University of Bologna, Bologna, Italy

I thank the Author and Editors for the opportunity to review this work. The straightforwardness of the mathematical approach is commendable as it provides clear numbers useful to formulate hypotheses on the main drivers of excess mortality. While the data extracted were age-standardized mortality rates, the most decisive limitations of observational studies persist.

 The Limitations section should include considerations on which biases (e.g. Chemaitelly et al. (2025) -Ref-1 ) and confounding factors (e.g. Acuti Martellucci et al. COVID-19 vaccination, all-cause mortality, and hospitalization for cancer: 30-month cohort study in an Italian province. EXCLI J. 2025 Jul 1;24:690-707. doi: 10.17179/excli2025-8400) may be at play. Also, while I acknowledge that Cox regression was not possible given the available dataset, the implications of a time dimension being absent from the analyses should be described (e.g. delayed diagnosis and treatment impacting vaccinated and unvaccinated differently?). Finally, the biological plausibility of a potential effect of vaccination on non-COVID mortality should be explored, albeit briefly. Once this is done, given the very early stage of research on this topic, I reckon that the language used in the Implications section and in the Abstract's Conclusions paragraph will have to be softened further. 

 On this note, the Abstract presents findings in a manner that is much too assertive given the methodological drawbacks. Actually, some results are presented in the Conclusions section, and some conclusions appear to be drawn in the Results section, hindering clarity. I would like to suggest some re-structuring, for instance as follows:

 1. for the Results section, eliminate any conclusive remarks, and possibly condense the second and third sentences of the Conclusions within the Results;

 2. for the Conclusions section, briefly summarize the main limitations of the work in one sentence, to highlight the (very) preliminary nature of the results, then follow with a simple explanation of what the main findings suggest (the suggestions for further research are appropriate).

 Finally, I am confused by the use of "increase (decrease)", "vaccinated (unvaccinated)", and "decrease (increase)" in the first paragraph of the Limitations section. Please either clarify or simplify to improve readability.

Is the work clearly and accurately presented and does it cite the current literature?

Partly

If applicable, is the statistical analysis and its interpretation appropriate?

Partly

Are all the source data underlying the results available to ensure full reproducibility?

Yes

Is the study design appropriate and is the work technically sound?

Yes

Are the conclusions drawn adequately supported by the results?

No

Are sufficient details of methods and analysis provided to allow replication by others?

Yes

Reviewer Expertise:

Epidemiology, Public Health

I confirm that I have read this submission and believe that I have an appropriate level of expertise to confirm that it is of an acceptable scientific standard, however I have significant reservations, as outlined above.

# Reviewer report: Reviewer response for version 5

**DOI:** 10.5256/f1000research.196436.r458573

**Published:** 2026-02-16

**Authors:** Mario Coccia

**Affiliations:** 1 Consiglio Nazionale delle Ricerche Area di Ricerca di Torino, Turin, Piedmont, Italy

I have read thoroughly the revised version of paper.

 Now this version of the paper after revision done is OK and provides interesting results for readers.

Is the work clearly and accurately presented and does it cite the current literature?

Partly

If applicable, is the statistical analysis and its interpretation appropriate?

Partly

Are all the source data underlying the results available to ensure full reproducibility?

Partly

Is the study design appropriate and is the work technically sound?

Partly

Are the conclusions drawn adequately supported by the results?

Partly

Are sufficient details of methods and analysis provided to allow replication by others?

Partly

Reviewer Expertise:

Pandemic preparedness, Pandemic crisis, COVID-19, Vaccination Policies, New technologies

I confirm that I have read this submission and believe that I have an appropriate level of expertise to confirm that it is of an acceptable scientific standard.

# Reviewer report: Reviewer response for version 4

**DOI:** 10.5256/f1000research.191164.r432356

**Published:** 2025-11-22

**Authors:** Mario Coccia

**Affiliations:** 1 Consiglio Nazionale delle Ricerche Area di Ricerca di Torino, Turin, Piedmont, Italy

Although minor changes, this version of the paper still provides interesting results for readers.

Is the work clearly and accurately presented and does it cite the current literature?

Partly

If applicable, is the statistical analysis and its interpretation appropriate?

Partly

Are all the source data underlying the results available to ensure full reproducibility?

Partly

Is the study design appropriate and is the work technically sound?

Partly

Are the conclusions drawn adequately supported by the results?

Partly

Are sufficient details of methods and analysis provided to allow replication by others?

Partly

Reviewer Expertise:

Pandemic preparedness, Pandemic crisis, COVID-19, Vaccination Policies

I confirm that I have read this submission and believe that I have an appropriate level of expertise to confirm that it is of an acceptable scientific standard.

# Reviewer report: Reviewer response for version 3

**DOI:** 10.5256/f1000research.188427.r420187

**Published:** 2025-11-08

**Authors:** Dan Romer

**Affiliations:** 1 University of Pennsylvania, Philadelphia, Pennsylvania, USA

The author wants to argue that protection due to vaccination was either temporary or increased the risk of mortality due to reasons other than Covid. However, using comparisons to mortality at the first month is extremely misleading (Figure 5)  because the unvaccinated had much higher rates of both Covid and nonCovid deaths at the outset of the pandemic, which declined over time, while the vaccinated did not change in their rates of nonCovid mortality. So, we are comparing two very different mortality patterns.

 The data show that the vaccinated were protected from Covid relative to the unvaccinated for an extended period, which appeared to end as the pandemic wore down. But this could be due to the protective effects of population immunity that grew over time. Without the vaccine, that protection would have taken longer to appear and would have led to more deaths at the beginning of the pandemic.

Is the work clearly and accurately presented and does it cite the current literature?

Partly

If applicable, is the statistical analysis and its interpretation appropriate?

No

Are all the source data underlying the results available to ensure full reproducibility?

Yes

Is the study design appropriate and is the work technically sound?

No

Are the conclusions drawn adequately supported by the results?

No

Are sufficient details of methods and analysis provided to allow replication by others?

Yes

Reviewer Expertise:

My main expertise is in vaccine protection at the population level.

I confirm that I have read this submission and believe that I have an appropriate level of expertise to state that I do not consider it to be of an acceptable scientific standard, for reasons outlined above.

# Response

**Published:** 2026-02-09

**Authors:** Jarle Aarstad

**Affiliations:** Western Norway University of Applied Sciences, Norway

Dear referee,

 Thanks for your constructive feedback on my manuscript, which I respond to below.

 You state that “using comparisons to mortality at the first month is extremely misleading (Figure 5) because the unvaccinated had much higher rates of both Covid and nonCovid deaths at the outset of the pandemic, which declined over time, while the vaccinated did not change in their rates of nonCovid mortality. So, we are comparing two very different mortality patterns.”

***Response:***

*It is correct that “the unvaccinated had much higher rates of both Covid and nonCovid deaths at the outset of the pandemic”, which I show in Figure 1. In other words, there is full transparency, and I hide nothing from the data.*

*From my reading of your report, we both seem to agree that the mortality of the unvaccinated “declined over time while the vaccinated did not change in their rates of nonCovid mortality.” This is evident in both Figure 1B and Figure 5. “[U]sing comparisons to mortality at the first month”, as I do in Figure 5, or showing both groups’ mortality rates per 100k as I do in Figure 1, does not change the core issue concerning the empirical findings. Both figures reveal the same pattern: “while mortality not involving COVID-19 decreased among unvaccinated compared to the first observation month, it remained high among vaccinated, indicating a relative increase” (quote from the abstract).*

*What is debatable, however, is WHY the morality declined among the unvaccinated but did not among the vaccinated, i.e., it remained relatively high. One reason for the decline among the unvaccinated is likely “mortality deficit”, due to two waves of excess mortality, shown in Figure 6, before the observations reported in Figures 1 and 5. In other words, the decline in mortality among the unvaccinated has a very good explanation.*

But why do we not observe a similar decline in mortality among the vaccinated? In Note 5, I address the following (in the revision that will be uploaded shortly, I have added some text to Note 5 in bold): “Assuming that the excess mortality among the unvaccinated segment **(those that would not opt for vaccination)** before Apr 21 was a percent, taking a positive value, one may assume that it was a*b percent among the vaccinated segment **(those that would opt for vaccination)**, where 0A parallel argument is that the excess mortality before Apr 21 cannot be attributed to the unvaccinated segment alone, as they only represent roughly 10% of the English population, ten years and older.” Taken together, one should expect “mortality deficit”, due to previous excess mortality, to induce a mortality decline also among the vaccinated, which we do not observe.

*The second issue is the continued excess mortality observed after Apr 2021 (Figure 6). Again, due to “mortality deficit”, i.e., two waves of excess mortality before that date, one should instead expect a decline, but this is only observed among the unvaccinated, a small fraction of 10% of the population. In other words, the excess mortality observed after 2021 can only be attributed to the vaccinated segment, which represents about 90% of the population, and shows a similar mortality pattern in Figures 1 and 5 to that in Figure 6 (while the mortality pattern among unvaccinated shows a dissimilar mortality pattern in Figures 1 and 5 to that in Figure 6).*

 Secondly, you state as follows: “The data show that the vaccinated were protected from Covid relative to the unvaccinated for an extended period, which appeared to end as the pandemic wore down. But this could be due to the protective effects of population immunity that grew over time. Without the vaccine, that protection would have taken longer to appear and would have led to more deaths at the beginning of the pandemic.”

***Response:***

*I agree that you have a point. I.e., population immunity from the majority of the population being vaccinated could also protect the unvaccinated, a form of indirect protection. However, early in 2022, Omicron became the dominant variant, for which vaccination provided limited indirect protection after three months* *(Tan et al., 2025)* *. In the revision that will be uploaded shortly, I have added the following statement to Note 3 (in bold): “One may attribute the non-significant effect from Feb 22 (Figure 2B) to relatively low mortality involving COVID-19 from that month (Figure 3). The reason for the argument is that the effects in Figure 2B would be absent if the mortality involving COVID-19 approached zero (which explains the non-significant effect between Apr and Jun 21). It is nonetheless worth noting, that among the vaccinated, the mortality rate in several months from Feb 22 was higher than in months between Jul 21 and Jan 22 (Figure 3). Hence, the decrease in mortality involving COVID-19 largely occurred among the unvaccinated. This is reflected in ORs of mortality involving COVID-19 among unvaccinated (compared to vaccinated as a reference group), decreasing from about 10 at the beginning to about 2 at the end (Figure 4). **One may further attribute the non-significant effect from Feb 22 (Figure 2B) to population immunity, as the majority of the population was vaccinated, protecting the unvaccinated, a form of indirect protection. However, from early 2022, Omicron became the dominant variant, for which vaccination provided limited indirect protection after three months*** ***(Tan et al., 2025)*** ***.”***

 New reference: 

 Tan, S. T., Rodríguez-Barraquer, I., Kwan, A. T., Blumberg, S., Park, H. J., Hutchinson, J., . . . Lo, N. C. (2025). Strength and durability of indirect protection against SARS-CoV-2 infection through vaccine and infection-acquired immunity. *Nat Commun, 16*(1), 1090. doi:10.1038/s41467-024-55029-9

# Reviewer report: Reviewer response for version 3

**DOI:** 10.5256/f1000research.188427.r423367

**Published:** 2025-11-07

**Authors:** Mina T Kelleni

**Affiliations:** 1 Pharmacology Department, Minia University, Minya, Menia Governorate, Egypt

I’d like to applaud the author for this interesting work to address a critical research gap in producing valid estimations when comparing non-randomized groups, specifically COVID-19 vaccinated versus unvaccinated populations using observational data. As someone who has closely studied the impact of vaccination policies, I recognize the difficulty and yet high importance of producing valid, unbiased estimates in non-randomized settings.

 Unlike many observational studies published during the July 2021 to January 2022 period that concluded vaccine protection against overall mortality, often while acknowledging but downplaying important limitations, this study presents a compelling alternative explanation. By methodically contrasting all-cause mortality with mortality not involving COVID-19, the author highlights that higher mortality among unvaccinated individuals at baseline likely reflects underlying health disparities rather than vaccine effects alone.

 Importantly, the critiques raised by an expert in epidemiology and mathematical modeling reviewer, if consistently applied, would have warranted rejection of those studies concluding the efficacy of COVID-19 vaccines in reducing the overall mortality at that period as well, which did not occur. This highlights an inconsistency in the evaluation process that underscores the importance of affording the author of this study a fair opportunity to present and substantiate his alternative viewpoint and analytical perspective. Given that seminal studies advocating vaccine efficacy faced similar methodological challenges but were nevertheless accepted, it is both reasonable and necessary to allow divergent scientific interpretations to be rigorously tested and debated within the literature. This approach fosters robust scientific discourse and protects the integrity of evidence synthesis, especially on issues as consequential as COVID-19 vaccination and mortality.

 Moreover, this study contributes to raising concerns about potential long-term adverse effects of COVID-19 vaccination, concerns that must not be overlooked or ignored as has unfortunately occurred with the short-term effects, which were often dismissed as "collateral damage" in the early phases of the pandemic response. These short-term adverse outcomes, particularly among otherwise healthy young individuals subjected to COVID-19 mandates, were minimized or marginalized in much of the mainstream discourse, leading to a lack of adequate acknowledgement and compensation, especially in low-resource settings.

 Overall, the author's simplified, exploratory, descriptive comparison method attempts to infer bias patterns in non-randomized data without using traditional covariate adjustment. It enriches the ongoing discourse on COVID-19 vaccine effectiveness and safety. It also fosters a more balanced understanding that considers both public health benefits and potential risks, underpinning the need for transparent, equitable policy decisions worldwide.

 Major remark:

 I’d like to advise the author to expand the discussion to address the above remarks, you may check [Reference 1], [Reference 2], [Reference 3] and their cited references as a starting point.

 Minor remarks:

 If possible, formulate a causal hypothesis regarding the effect of vaccination on mortality. Develop a Directed Acyclic Graph (DAG) illustrating hypothesized relationships among vaccination, mortality outcomes, and key confounders (such as comorbidities, socioeconomic status, age, sex, and ethnicity).

 If accessible, incorporate individual-level data to implement advanced confounder adjustment techniques such as Propensity Score Matching (PSM) or Inverse Probability Weighting (IPW). If data limitations prevent this, explicitly acknowledge this restriction.

 If feasible, segregate analysis periods based on dominant SARS-CoV-2 variants (e.g., Alpha/Delta vs. Omicron) to account for changing epidemic dynamics. Consider time-dependent modeling approaches (e.g., Cox models with time-varying covariates) to more accurately capture the evolving context.

 If possible, implement competing-risk models to distinguish COVID-19 from non-COVID mortality and conduct stratified analyses across age groups, vaccine types, dose numbers, and intervals since vaccination to elucidate heterogeneity.

Is the work clearly and accurately presented and does it cite the current literature?

Yes

If applicable, is the statistical analysis and its interpretation appropriate?

Partly

Are all the source data underlying the results available to ensure full reproducibility?

Partly

Is the study design appropriate and is the work technically sound?

Yes

Are the conclusions drawn adequately supported by the results?

Yes

Are sufficient details of methods and analysis provided to allow replication by others?

Partly

Reviewer Expertise:

COVID-19 and COVID-19 vaccines immunopharmacology and immunotoxicology.

I confirm that I have read this submission and believe that I have an appropriate level of expertise to confirm that it is of an acceptable scientific standard, however I have significant reservations, as outlined above.

## References

[1] : What would Hippocrates have sworn upon witnessing the COVID-19 mandates and mortality paradox. *World Journal of Experimental Medicine* .2025;15(1) : 10.5493/wjem.v15.i1.98575 10.5493/wjem.v15.i1.98575 PMC1171858940115753

[2] : COVID-19 mortality paradox (United Statesvs Africa): Mass vaccinationvs early treatment. *World Journal of Experimental Medicine* .2024;14(1) : 10.5493/wjem.v14.i1.88674 10.5493/wjem.v14.i1.88674 PMC1099906938590304

[3] : SARS CoV-2 Vaccination Autoimmunity, Antibody Dependent Covid-19 Enhancement and Other Potential Risks: Beneath the Tip of the Iceberg. *International Journal of Pulmonary & Respiratory Sciences* .2021;5(2) : 10.19080/IJOPRS.2021.05.555658 10.19080/IJOPRS.2021.05.555658

# Response

**Published:** 2025-11-08

**Authors:** Jarle Aarstad

**Affiliations:** Western Norway University of Applied Sciences, Norway

#1. Referee comment:

 I’d like to applaud the author for this interesting work to address a critical research gap in producing valid estimations when comparing non-randomized groups, specifically COVID-19 vaccinated versus unvaccinated populations using observational data. As someone who has closely studied the impact of vaccination policies, I recognize the difficulty and yet high importance of producing valid, unbiased estimates in non-randomized settings.

 Unlike many observational studies published during the July 2021 to January 2022 period that concluded vaccine protection against overall mortality, often while acknowledging but downplaying important limitations, this study presents a compelling alternative explanation. By methodically contrasting all-cause mortality with mortality not involving COVID-19, the author highlights that higher mortality among unvaccinated individuals at baseline likely reflects underlying health disparities rather than vaccine effects alone.

 Importantly, the critiques raised by an expert in epidemiology and mathematical modeling reviewer, if consistently applied, would have warranted rejection of those studies concluding the efficacy of COVID-19 vaccines in reducing the overall mortality at that period as well, which did not occur. This highlights an inconsistency in the evaluation process that underscores the importance of affording the author of this study a fair opportunity to present and substantiate his alternative viewpoint and analytical perspective. Given that seminal studies advocating vaccine efficacy faced similar methodological challenges but were nevertheless accepted, it is both reasonable and necessary to allow divergent scientific interpretations to be rigorously tested and debated within the literature. This approach fosters robust scientific discourse and protects the integrity of evidence synthesis, especially on issues as consequential as COVID-19 vaccination and mortality.

 Moreover, this study contributes to raising concerns about potential long-term adverse effects of COVID-19 vaccination, concerns that must not be overlooked or ignored as has unfortunately occurred with the short-term effects, which were often dismissed as "collateral damage" in the early phases of the pandemic response. These short-term adverse outcomes, particularly among otherwise healthy young individuals subjected to COVID-19 mandates, were minimized or marginalized in much of the mainstream discourse, leading to a lack of adequate acknowledgement and compensation, especially in low-resource settings.

 Overall, the author's simplified, exploratory, descriptive comparison method attempts to infer bias patterns in non-randomized data without using traditional covariate adjustment. It enriches the ongoing discourse on COVID-19 vaccine effectiveness and safety. It also fosters a more balanced understanding that considers both public health benefits and potential risks, underpinning the need for transparent, equitable policy decisions worldwide.

 Author response:

 Thank you for this positive feedback. I agree with you, and your note is a valuable contribution to the discourse on the study’s topic.

 #2. Referee comment:

 Major remark:

 I’d like to advise the author to expand the discussion to address the above remarks, you may check [Reference 1], [Reference 2], [Reference 3] and their cited references as a starting point.

 References

 1. Kelleni M: What would Hippocrates have sworn upon witnessing the COVID-19 mandates and mortality paradox. World Journal of Experimental Medicine. 2025; 15 (1). Publisher Full Text

 2. Kelleni M: COVID-19 mortality paradox (United Statesvs Africa): Mass vaccinationvs early treatment. World Journal of Experimental Medicine. 2024; 14 (1). Publisher Full Text

 Author response:

 The revision includes the following edited sentence in the Discussion with the suggested references (along with other references as you will find in the text):

 Finally, as the study indicated that COVID-19 vaccination may have increased mortality in a longer perspective, it contributes to and aligns with other research that also shows the intervention can have adverse effects (1) and increase mortality, including from the virus (2, 3).

 1.         Kelleni MT. SARS-CoV-2 vaccination, autoimmunity, antibody dependent Covid-19 enhancement and other potential risks: Beneath the tip of the iceberg. Int J Pulm Respir Sci. 2021;5(2):1–10.

 2.         Kelleni MT. What would Hippocrates have sworn upon witnessing the COVID-19 mandates and mortality paradox. World J Exp Med. 2025;15(1):98575.

 3.         Kelleni MT. COVID-19 mortality paradox (United States vs Africa): Mass vaccination vs early treatment. World J Exp Med. 2024;14(1):88674.

 #3. Referee comment:

 Minor remarks:

 If possible, formulate a causal hypothesis regarding the effect of vaccination on mortality. Develop a Directed Acyclic Graph (DAG) illustrating hypothesized relationships among vaccination, mortality outcomes, and key confounders (such as comorbidities, socioeconomic status, age, sex, and ethnicity).

 Author response:

 Referee 2 also suggested that I should formulate hypotheses, to which I responded in the previous report:

 I agree that postulating one or more hypotheses in a deductive setting has advantages, which are tested in, for instance, regression models providing significant or non-significant empirical findings. However, I cannot see how I can study my research question using that approach, as I consider the examination of my research question to be more of a puzzle where different analyses (graphed in the different figures) in totality provide information about what the overall empirical landscape looks like. My general research question is as follows (p. 3): “how do the mortality patterns differ in England from Apr 21 to May 23 between COVID-19 vaccinated and unvaccinated?”, and I argue that my study illuminates that research question fairly well. “The study’s major contribution is to illustrate how contrasting all-cause mortality with mortality not involving COVID-19 may indicate valid estimates between non-randomized groups of vaccinated and unvaccinated.” To do that, I further discuss data on mortality involving COVID-19. … I argue that my empirical data and the explanations of them sensibly address the research question, but without overexplaining the interpretations.

 The issues addressed above suggest that formulating hypotheses and developing a Directed Acyclic Graph are not feasible in the current study, I argue.

 #4. Referee comment:

 If accessible, incorporate individual-level data to implement advanced confounder adjustment techniques such as Propensity Score Matching (PSM) or Inverse Probability Weighting (IPW). If data limitations prevent this, explicitly acknowledge this restriction.

 Author response:

 Only age-standardized data is available, yet in the study I emphasize the following:

 Introduction (with appropriate references partly omitted here): Variables accounting for potentially confounding effects are often unavailable or unknown, and including those available but unknowingly improper can increase bias. In line with the reasoning, York showed that “unless *all* potential confounding factors are included in an analysis (which is unlikely to be achievable with most real-world data-sets), adding control variables to a model in many circumstances can make estimated effects … less accurate.” I argue there is a research gap concerning valid estimations between non-randomized groups, such as COVID-19 vaccinated and unvaccinated, which is challenging even when including seemingly relevant control variables that can actually deteriorate the results. 7 To address the research gap, using English data covering 26 months from Apr 21 to May 23, 9 I explain an achievable approach by contrasting all-cause mortality among COVID-19 vaccinated and unvaccinated with mortality not involving COVID-19 among COVID-19 vaccinated and unvaccinated.

 Discussion: As most previous studies on the link between COVID-19 vaccination and mortality have been carried out in non-randomized contexts and, accordingly, even in the presence of control variables exposed to validity concerns, this study has illustrated and applied a useful tool to address those limitations. Moreover, I argue that the tool has general applicability as it can also be used in other research contexts.

 #5. Referee comment:

 If feasible, segregate analysis periods based on dominant SARS-CoV-2 variants (e.g., Alpha/Delta vs. Omicron) to account for changing epidemic dynamics. Consider time-dependent modeling approaches (e.g., Cox models with time-varying covariates) to more accurately capture the evolving context.

 Author response:

 I did not have access to data on dominant variants. From my perspective, I could not apply a Cox model because the data did not provide information on the time to an event. Instead, the data provided information about the number of deaths at different time points, which enabled logistic regression to be an appropriate application.

 #5. Referee comment:

 If possible, implement competing-risk models to distinguish COVID-19 from non-COVID mortality and conduct stratified analyses across age groups, vaccine types, dose numbers, and intervals since vaccination to elucidate heterogeneity.

 Author response:

 I agree that those topics are relevant, but including all of them would significantly expand the paper. In the Limitations and future research section, I therefore write as follows:

 This study included those ten years and older. I, therefore, encourage future research to analyze different age cohorts separately to assess how findings may converge or eventually diverge. As this study merely distinguished between those vaccinated and those who were not, I also encourage future research to distinguish between those who received one or more doses and different vaccine types, although it may be methodologically challenging.

 #6. Referee comment:

 If possible, implement competing-risk models to distinguish COVID-19 from non-COVID mortality and conduct stratified analyses across age groups, vaccine types, dose numbers, and intervals since vaccination to elucidate heterogeneity.

 Author response:

 I agree that those topics are relevant, but including all of them would significantly expand the paper. In the Limitations and future research section, I therefore write as follows:

 This study included those ten years and older. I, therefore, encourage future research to analyze different age cohorts separately to assess how findings may converge or eventually diverge. As this study merely distinguished between those vaccinated and those who were not, I also encourage future research to distinguish between those who received one or more doses and different vaccine types, although it may be methodologically challenging.

 #7. Referee comment:

 Is the work clearly and accurately presented and does it cite the current literature?

 Yes

 Author response:

 Thanks for your acknowledgement.

 #8. Referee comment:

 Is the study design appropriate and is the work technically sound?

 Yes

 Author response:

 Thanks for your acknowledgement.

 #9. Referee comment:

 Are sufficient details of methods and analysis provided to allow replication by others?

 Partly

 Author response:

 First, data is publicly available, and I present appropriate references (please see #11). Second, I have reread the Methods and Results section to ensure that all necessary information is given there to enable replication of the analyses.

 #10. Referee comment:

 If applicable, is the statistical analysis and its interpretation appropriate?

 Partly

 Author response:

 I hope my comments regarding #5 and #6 adequately address this issue.

 #11. Referee comment:

 Are all the source data underlying the results available to ensure full reproducibility?

 Partly

 Author response:

 Please read what I write concerning data availability:

 UK Office for National Statistics (reference) Deaths by vaccination status, England 2023: https://www.ons.gov.uk/peoplepopulationandcommunity/birthsdeathsandmarriages/deaths/datasets/deathsbyvaccinationstatusengland I used the dataset labeled “Deaths occurring between 1 April 2021 and 31 May 2023 edition of this dataset”, Table 1: Unvaccinated and Ever vaccinated. The Methods section explains in detail how I modeled the data.

 #12. Referee comment:

 Are the conclusions drawn adequately supported by the results?

 Yes

 Author response:

 Thanks for your acknowledgement.

# Reviewer report: Reviewer response for version 2

**DOI:** 10.5256/f1000research.179445.r375386

**Published:** 2025-09-01

**Authors:** Gregory Barnsley

**Affiliations:** 1 London School of Hygiene and Tropical Medicine, London, UK

The new version of this report includes several changes that improve the clarity and highlight some limitations. However, the report contains several errors and does not do a convincing job of demonstrating the validity of its methods or through reasoning for its conclusions. In short, it concludes too much from too little.

 Major Issues with methodology:

 The new section in the methods is incorrect. Assume that the rate of deaths is Mv (total mortality in the vaccinated), Mu (mortality in the unvaccinated), Nv (non-COVID in the vaccinated), Nu, Cv (COVID mortality in the vaccinated), Cu (COVID mortality in the unvaccinated). Then let Mu be 60% greater than Mv (Mu/Mv = 1.6). Let the non-COVID mortality be (1-k)%, then Nu/Nv = k. We also have that Mv = Nv + Cv and Mu = Nu + Cu. The relative mortality of COVID in the unvaccinated is Cu/Cv. Now, if non-COVID mortality is 60% greater in the unvaccinated (k = 1.6,) then we get Cu/Cv = (Mu - Nu)/(Mv - Nv) = (1.6Mv - kNv)/(Mv - Nv) = 1.6. Hence, it does not imply that vaccinations don’t provide protection. If we solve for k, we find that k = (0.6Mv + Nv)/Nv for the COVID mortality in the two groups to be the same. The other numbers given in the example also do not hold. This also undermines the point made when referring to this argument with Fig 2A; however, this is also not that relevant since these rates (or odds ratios) are not directly compared that way.

 The reasoning around when non-COVID mortality rates are equal does not make sense either; you could assume that the unvaccinated are unhealthy in a way that greatly increases the risk of COVID-19 death and not non-COVID deaths (though crudely this would be indistinguishable from a vaccine effect).

 I still do not see how the rate transformations are necessary. The transformed data would still not give “correct” logistic ORs since they are constructed using age-adjusted rates. Why can’t you just compare the rates using the person-years given, without the transformations?

 There is still almost no consideration of other factors that might explain the observed trends. The author concludes that the limited duration of vaccine protection (against COVID deaths) is evidence of the unhealthy vaccine effect but that could equally be due to the rise of COVID variants (such as Omicron that arose shortly before the relevant period, with evidence of reduced mortality and reduced vaccine efficacy) or other changes to behaviour over this time.

 The author makes some attempt to dismiss the limitation that the unvaccinated population could have started unhealthy, but on an aggregate level, improved in health (due to deaths, vaccination or behaviour change). In general, rates in risk groups may be lower, but maybe not on the scale at which this data is presented (i.e. by month), but the author does not explore this data.

 The fact that apparent high-risk groups have higher rates of non-vaccination is alluded to in this paper with no in-depth exploration. While the author acknowledges that improper adjustment for confounders can bias results further, they make a worse mistake by adjusting for these variables in their mental model in a way that cannot be quantified and hence cannot be examined. This holds for the fact that they use age-adjusted death rates; is that not exactly the partial confounding that the author cautions against in the introduction?

 In general, the report is unconvincing and concludes too much from its data. The author should indicate what their hypothesis is and what we would expect to see in the observations based on this. They should also indicate where these observations would contradict other, more common explanations. However, I don’t believe this is viable from such a limited set of data (particularly with no exploration of potential confounders), the author should greatly limit their conclusions.

 Finally, a couple of minor points:

 Wouldn’t it be viable to do a sensitivity analysis, including the other ICD codes, and see how that impacts the results?

 Figures 5/6 are only mentioned in notes, which is confusing; they could be addressed in the discussion instead.

Is the work clearly and accurately presented and does it cite the current literature?

Partly

If applicable, is the statistical analysis and its interpretation appropriate?

No

Are all the source data underlying the results available to ensure full reproducibility?

Yes

Is the study design appropriate and is the work technically sound?

Partly

Are the conclusions drawn adequately supported by the results?

No

Are sufficient details of methods and analysis provided to allow replication by others?

Yes

Reviewer Expertise:

Epidemiology and mathematic modelling. I am not a demographer so I cannot comment on any particularities of mortality rates.

I confirm that I have read this submission and believe that I have an appropriate level of expertise to state that I do not consider it to be of an acceptable scientific standard, for reasons outlined above.

# Response

**Published:** 2025-09-18

**Authors:** Jarle Aarstad

**Affiliations:** Western Norway University of Applied Sciences, Norway

Dear Referee 2,

 I greatly appreciate the time and effort you took to provide critical, yet constructive, feedback on my previous version of the paper. In the following, you will read how I have addressed your comments. For your information, I have also made minor corrections and edits in the text to improve accuracy and readability. 

 Sincerely,

 The author.

 Comment #1

 The new version of this report includes several changes that improve the clarity and highlight some limitations. However, the report contains several errors and does not do a convincing job of demonstrating the validity of its methods or through reasoning for its conclusions. In short, it concludes too much from too little.

 Response #1

 In the following, you will read my responses to the specific issues you have raised.

 Comment #2

 Major Issues with methodology:

 The new section in the methods is incorrect. Assume that the rate of deaths is Mv (total mortality in the vaccinated), Mu (mortality in the unvaccinated), Nv (non-COVID in the vaccinated), Nu, Cv (COVID mortality in the vaccinated), Cu (COVID mortality in the unvaccinated). Then let Mu be 60% greater than Mv (Mu/Mv = 1.6). Let the non-COVID mortality be (1-k)%, then Nu/Nv = k. We also have that Mv = Nv + Cv and Mu = Nu + Cu. The relative mortality of COVID in the unvaccinated is Cu/Cv. Now, if non-COVID mortality is 60% greater in the unvaccinated (k = 1.6,) then we get Cu/Cv = (Mu - Nu)/(Mv - Nv) = (1.6Mv - kNv)/(Mv - Nv) = 1.6. Hence, it does not imply that vaccinations don’t provide protection. If we solve for k, we find that k = (0.6Mv + Nv)/Nv for the COVID mortality in the two groups to be the same. The other numbers given in the example also do not hold.

 Response #2

 I acknowledge your math. Therefore, I have omitted the discussion you referred to in the revision. Additionally, I have done my best to address your comment in the revised version, taking it into account when presenting my findings. Please see my responses below. 

 Comment #3

 This also undermines the point made when referring to this argument with Fig 2A; however, this is also not that relevant since these rates (or odds ratios) are not directly compared that way.

 Response #3

 Related to Fig. 2A (p. 6), I write as follows: “At the beginning of the period, the ORs of all-cause mortality (marked in green) among unvaccinated were approximately between 2 and 2.5 compared to vaccinated (significant at the 95% CIs), and mortality not involving COVID-19 (marked in orange) shows a similar pattern.” I assume we can agree on that statement.

 Next, I write: “In parallel, Figure 3 shows that the mortality rate involving COVID-19 was low at the beginning of the period for both vaccinated and unvaccinated (A and B are identical, except for different scaling).” [Figure 3 was Figure 5 in the previous version]. Also, I assume we can agree on that statement.

 Drawing an implication of what I write above, I continue as follows: “Therefore, I conclude that unvaccinated had between 2 and 2.5 times higher ORs of all-cause mortality and mortality not involving COVID-19 compared to vaccinated at the beginning of the period, largely due to inferior health at the outset, and not vaccine protection since the overall mortality involving COVID-19 during that period was low. The argument is grounded in the assumption that the vaccine unlikely protects against mortality not involving COVID-19. 26 That is, if close to zero people died from COVID-19, I cannot see any logical reason why the mortality pattern observed at the beginning of the period has another explanation than unvaccinated having inferior health at the outset.” I do hope we can agree on the arguments that I have addressed here.

 Also, I hope we can agree on the following statement, which, from my point of view does not contradict your mathematical explanation: “Between the last half of 21 and the beginning of 22, on the other hand, the ORs were higher for all-cause mortality than for mortality not involving COVID-19 (Figure 2A), which may indicate a temporal preventive vaccine effect. Figure 3 supports that assumption as it particularly shows an uptick in the mortality rate involving COVID-19 among unvaccinated during that period. However, we cannot rule out that the uptick may not be due to vaccine protection, but instead high vulnerability at the outset among unvaccinated to die from the virus infection. Yet an argument countering that assumption is that the ORs of mortality involving COVID-19 among unvaccinated (compared to vaccinated as a reference group), although significant during the whole period (95% CIs), were down from about 10 at the beginning to about 2 at the end (Figure 4). The decrease may either indicate temporal but declining vaccine protection, potentially because of the rise of the Omicron variant, or the relative increase in mortality among vaccinated may indicate a detrimental health effect, which I address below [in the article].”

 Concerning ORs, please see #5.

 Comment #4

 The reasoning around when non-COVID mortality rates are equal does not make sense either; you could assume that the unvaccinated are unhealthy in a way that greatly increases the risk of COVID-19 death and not non-COVID deaths (though crudely this would be indistinguishable from a vaccine effect).

 Response #4

 (i) I agree “that you could assume that the unvaccinated are unhealthy in a way that greatly increases the risk of COVID-19 death”, which I particularly illustrate in Figure 3 (Figure 5 in the previous version) and address in the manuscript (please see #3). In the revision, I cannot see that it contradicts your argument.

 (ii) You also state that “you could assume that the unvaccinated are unhealthy in a way that [does not increase] non-COVID deaths”. Theoretically, your statement may be correct, even though I do not find it very plausible from a medical point of view. Nonetheless, I argue that the data I analyze falsify your statement, the reason being that all-cause mortality and mortality not involving COVID-19 were much higher among the unvaccinated compared to the vaccinated at the beginning of the period, when registered COVID-19-related deaths were very low (please see #3).

 Finally, from my understanding, you argue that the above issues crudely “would be indistinguishable from a vaccine effect”. First, from my point of view, I show that (ii) is distinguishable from a vaccine effect as all-cause mortality and mortality not involving COVID-19 were much higher among unvaccinated compared to vaccinated at the beginning of the period when registered COVID-19 related deaths were very low. Concerning (i), I acknowledge that you have a valid point. In the revision, I therefore write as follows (p. 6): “we cannot rule out that the uptick [in mortality involving COVID-19, particularly among unvaccinated] may not be due to vaccine protection, but instead high vulnerability at the outset among unvaccinated to die from the virus infection. Yet an argument countering that assumption is that the ORs of mortality involving COVID-19 among unvaccinated (compared to vaccinated as a reference group), although significant during the whole period (95% CIs), were down from about 10 at the beginning to about 2 at the end (Figure 4). The decrease may either indicate temporal but declining vaccine protection, potentially because of the rise of the Omicron variant, or the relative increase in mortality among vaccinated may indicate a detrimental health effect, which I address below. To summarize, the vaccine may have provided temporary but declining protection, but we cannot rule out an increasingly detrimental health effect among vaccinated as an alternative or complementary explanation.”

 Comment #5

 I still do not see how the rate transformations are necessary. The transformed data would still not give “correct” logistic ORs since they are constructed using age-adjusted rates. Why can’t you just compare the rates using the person-years given, without the transformations?

 Response #5

 I agree that the transformation I report on in Figure 2B may be redundant. In the revision, I accordingly write as follows: “Substantially, Figure 2A and Figure 2B provide the same information …, but in my opinion, the latter illuminates the contrast between all-cause mortality and mortality not involving COVID-19 better...”

 Stating that “The transformed data would still not give ‘correct’ logistic ORs since they are constructed using age-adjusted rates” in my opinion would imply that the age-adjusted rates are also invalid. On the other hand, if the age-adjusted rates provide a valid picture, then the transformed logistic ORs would also provide an equally valid picture. Moreover, from my reading, it appears that age-adjusted ORs ratios have also been reported in other research (e.g., https://cardiab.biomedcentral.com/articles/10.1186/s12933-020-01159-5). In itself, that does not suffice to defend my approach, but I cannot see how my approach provides substantially uninformative ORs. If yes, from my understanding, the mortality ratios would be equally uninformative.

 In addition, from my perspective, the ORs in Figure 2A provide more precise information about mortality (all-cause and mortality not involving COVID-19) among the unvaccinated compared to the vaccinated, which is not as evident in Figures 1A and 1B. Similarly, I argue that ORs in Figure 4 give more precise information about mortality involving COVID-19 among unvaccinated compared vaccinated than what we observe in Figure 3. E.g., ORs being reduced from about 10 to 2 in Figure 4 is not easily observable in Figure 3.

 Comment #6

 There is still almost no consideration of other factors that might explain the observed trends. The author concludes that the limited duration of vaccine protection (against COVID deaths) is evidence of the unhealthy vaccine effect but that could equally be due to the rise of COVID variants (such as Omicron that arose shortly before the relevant period, with evidence of reduced mortality and reduced vaccine efficacy) or other changes to behaviour over this time.

 Response #6

 First, at least in the revision, I do not conclude “that the limited duration of vaccine protection (against COVID deaths) is evidence of the unhealthy vaccine effect”. Instead, I conclude “that vaccination, despite a potential temporary protection, may have increased mortality” (p. 1). In other words, a very tentative conclusion. I do not use the phrase “evidence” a single time. Nor, as far as I can see, do I use similar phrases. Concerning the “consideration of other factors that might explain the observed trends”, I believe I make sober reflections in “Limitations and future research”. Also, I address similar issues in Note 3. The arise of Omicron may address the fall ORs in Table 4, which I address in the revision, writing in relationship to Figure 4 (p. 6): “The decrease [in ORs of mortality involving COVID-19 among unvaccinated (compared to vaccinated as a reference group)] may either indicate temporal but declining vaccine protection, potentially because of the rise of the Omicron variant, or the relative increase in mortality among vaccinated may indicate a detrimental health effect, which I address below [in the paper].” Concerning Figure 5 (Figure 3 in the previous version), I cannot see that the Omicron variant may have had a substantial impact, as it includes mortality data not involving COVID-19 only.

 Comment #7

 The author makes some attempt to dismiss the limitation that the unvaccinated population could have started unhealthy, but on an aggregate level, improved in health (due to deaths, vaccination or behaviour change). In general, rates in risk groups may be lower, but maybe not on the scale at which this data is presented (i.e. by month), but the author does not explore this data.

 Response #7

 I assume you here refer to the data I present in Figure 5 (Figure 3 in the previous version) of mortality not involving COVID-19, and addressing the limitation concerning that “During the study period, a share of people in the unvaccinated group were transferred to the vaccinated” (p. 11). However, I cannot see that I attempted “to dismiss the limitation that the unvaccinated population could have started unhealthy”. Instead, I showed (in Figure 5) “that while mortality not involving COVID-19 decreased among unvaccinated (marked in red) compared to the first observation month, it remained high among vaccinated (marked in blue)”. In my opinion, this is an undisputable empirical observation, and as long as accounting for potential limitation concerning the dynamics in the unvaccinated vs. unvaccinated cohorts (which I believe I addressed adequately on p. 8 and in Note 5), one may therefore conclude as I do: “the data show a relatively high and relative increase in mortality not involving COVID-19 among vaccinated. An interpretation may be that vaccination, despite temporary protection, increased mortality. Strengthening the interpretation was relatively high mortality among vaccinated not involving COVID-19 counterintuitively following periods of excess mortality (Figure 6) …. Further strengthening the interpretation was the relatively high mortality not involving COVID-19 among the vaccinated, corresponding with excess mortality during much of the same period (ibid.) ….”  

 Comment #8

 The fact that apparent high-risk groups have higher rates of non-vaccination is alluded to in this paper with no in-depth exploration. While the author acknowledges that improper adjustment for confounders can bias results further, they make a worse mistake by adjusting for these variables in their mental model in a way that cannot be quantified and hence cannot be examined. This holds for the fact that they use age-adjusted death rates; is that not exactly the partial confounding that the author cautions against in the introduction?

 Response #8

 You state that “The fact that apparent high-risk groups have higher rates of non-vaccination is alluded to in this paper with no in-depth exploration.” In my opinion, I addressed the issue adequately. First, I related the statement by the UK Office for National Statistics, “rates for COVID-19 unvaccinated adults in England ‘were higher for Black Caribbean, Black African and White Other ethnic groups. Rates were also higher for those living in deprived areas, who have never worked or are long-term unemployed, who are limited a lot by a disability, … or who are male’”, to vaccine hesitancy research (with proper references). Then, I state that the above citation from the UK Office for National Statistics “indicates that unvaccinated have inferior health at the outset compared to vaccinated, inducing biased comparisons as the groups are not randomly assigned.” In the paper’s empirical section, I further address the issue related to the likely difference between non-randomized groups in much detail, as far as I can see.

 Then you state that “While the author acknowledges that improper adjustment for confounders can bias results further, they make a worse mistake by adjusting for these variables in their mental model in a way that cannot be quantified and hence cannot be examined. This holds for the fact that they use age-adjusted death rates; is that not exactly the partial confounding that the author cautions against in the Introduction?” Ok, it seems that we agree on the issue “that improper adjustment for confounders can bias results further”, but York states that “unless all potential confounding factors are included in an analysis (which is unlikely to be achievable with most real-world data-sets), adding control variables to a model in many circumstances can make estimated effects … less accurate” (cited on p. 2 in my paper). He does say that adding any control variable will, but can, make the estimates “less accurate”. As the data I apply match for age, theoretically, we can therefore assume that the estimates are less accurate, but I cannot see any logical reason for that. However, on the contrary, assuming that matching for age were to increase bias, I would still argue that the way I interpret the data would yield a similar conclusion, the reason being that, whether matching or not matching for age, one could nonetheless expect that vaccinated and non-vaccinated are dissimilar at the outset concerning health profile.

 Comment #9

 In general, the report is unconvincing and concludes too much from its data. The author should indicate what their hypothesis is and what we would expect to see in the observations based on this. They should also indicate where these observations would contradict other, more common explanations. However, I don’t believe this is viable from such a limited set of data (particularly with no exploration of potential confounders), the author should greatly limit their conclusions.

 Response #9

 I hope that my revisions, on which I have commented above, clarify the study’s contribution. What I can conclude from the data, and what I cannot. I agree that the previous version may have drawn too many conclusions from the data. In the revision, I have accordingly applied wordings such as follows:

 “First, I found that all-cause mortality among unvaccinated was higher than among vaccinated. [I believe that statement is indisputable.] But, as the pattern was similar concerning mortality not involving COVID-19, the discrepancy may be attributed mainly to unvaccinated having inferior health at the outset” (p. 1) [Note that I write “may”, but having said that, I believe the finding has relatively strong empirical support due to my findings, and om which I report (p. 6): I conclude that unvaccinated had between 2 and 2.5 times higher ORs of all-cause mortality and mortality not involving COVID-19 compared to vaccinated at the beginning of the period, largely due to inferior health at the outset, and not vaccine protection since the overall mortality involving COVID-19 during that period was low. The argument is grounded in the assumption that the vaccine unlikely protects against mortality not involving COVID-19. That is, if close to zero people died from COVID-19, I cannot see any logical reason why the mortality pattern observed at the beginning of the period has another explanation than unvaccinated having inferior health at the outset.”]

 Then I write that “There were nonetheless indications of significant protection for vaccinated between July 21 and Jan 22” (p. 1) [Note that I write “indications of significant protection…”. My statement is grounded in how I address the presentation and discussion of the relevant data (p. 6): “Between the last half of 21 and the beginning of 22 … the ORs were higher for all-cause mortality than for mortality not involving COVID-19 (Figure 2A), which may indicate a temporal preventive vaccine effect. Figure 3 supports that assumption as it particularly shows an uptick in the mortality rate involving COVID-19 among unvaccinated during that period. However, we cannot rule out that the uptick may not be due to vaccine protection, but instead high vulnerability at the outset among unvaccinated to die from the virus infection. Yet an argument countering that assumption is that the ORs of mortality involving COVID-19 among unvaccinated (compared to vaccinated as a reference group), although significant during the whole period (95% CIs), were down from about 10 at the beginning to about 2 at the end (Figure 4). The decrease may either indicate temporal but declining vaccine protection, potentially because of the rise of the Omicron variant, or the relative increase in mortality among vaccinated may indicate a detrimental health effect…. To summarize, the vaccine may have provided temporary but declining protection, but we cannot rule out an increasingly detrimental health effect among vaccinated as an alternative or complementary explanation.”]

 Finally, I write (p. 1): “while mortality not involving COVID-19 decreased among unvaccinated compared to the first observation month, it was high among vaccinated, indicating a relative increase”. I hope we can agree on that statement.

 From my point of view, in the revision, I have done my utmost not to draw more conclusions from the data than what is reasonably plausible.

 You state that “The author should indicate what their hypothesis is and what we would expect to see in the observations based on this.” I agree that postulating one or more hypotheses in a deductive setting has advantages, which are tested in, for instance, regression models providing significant or non-significant empirical findings. However, I cannot see how I can study my research question using that approach, as I consider the examination of my research question to be more of a puzzle where different analyses (graphed in the different figures) in totality provide information about what the overall empirical landscape looks like. My general research question is as follows (p. 3): “how do the mortality patterns differ in England from Apr 21 to May 23 between COVID-19 vaccinated and unvaccinated?”, and I argue that my study illuminates that research question fairly well. “The study’s major contribution is to illustrate how contrasting all-cause mortality with mortality not involving COVID-19 may indicate valid estimates between non-randomized groups of vaccinated and unvaccinated.” To do that, I further discuss data on mortality involving COVID-19. From my writings above (particularly #3 and #4), in my opinion, I argue that my empirical data and the explanations of them sensibly address the research question, but without overexplaining the interpretations. 

 You continue writing, “They [the hypotheses] should also indicate where these observations would contradict other, more common explanations.” In my opinion, I argue that my research question and postulated contribution explain how I contribute to the current research literature. In the Discussion (p. 8), I explain in detail how studying my research question aligns with and contributes to the existing research literature.

 Finally, you write that “I don’t believe this is viable from such a limited set of data (particularly with no exploration of potential confounders), the author should greatly limit their conclusions.” In my opinion, I argue that this revised version, in particular, provides a sober interpretation of the available and analyzed data. Moreover, the issue of “confounders”, which I admit were available for the data I analyzed, can, even in their presence, be challenging concerning validity, as I address in the paper’s Introduction.

 Comment #10

 Finally, a couple of minor points:

 Wouldn’t it be viable to do a sensitivity analysis, including the other ICD codes, and see how that impacts the results?

 Response #10

 Unfortunately, I do not have access to the data you refer to. Addressing limitations, I write as follows: “The validity of the finding indicating that vaccinated had significant protection between July 21 and Jan 22 hinges on non-systematic skewness in classifying false positives concerning mortality involving COVID-19 and false negatives concerning mortality not involving COVID-19.” I further elaborate on that topic in the manuscript.

 Comment #11

 Figures 5/6 are only mentioned in notes, which is confusing; they could be addressed in the discussion instead.

 Response #11

 In the revision, I have particularly addressed Figure 5 (Figure 3 in the revision) when presenting the results. The same goes for Figure 6 (Figure 4 in the revision). Please also see #3.

# Reviewer report: Reviewer response for version 2

**DOI:** 10.5256/f1000research.179445.r375385

**Published:** 2025-04-11

**Authors:** Mario Coccia

**Affiliations:** 1 Consiglio Nazionale delle Ricerche Area di Ricerca di Torino, Turin, Piedmont, Italy

I have read thoroughly the revised version of paper.

 The authors have done considerable additional work, and addressed all concerns and criticisms in the revised manuscript, which I believe has improved substantially in the theoretical framework, study design and discussion of results.

 Now, the paper is OK and has a good level to show interesting results to scholars and/or policymakers interested in these topics.

Is the work clearly and accurately presented and does it cite the current literature?

Partly

If applicable, is the statistical analysis and its interpretation appropriate?

Partly

Are all the source data underlying the results available to ensure full reproducibility?

Partly

Is the study design appropriate and is the work technically sound?

Partly

Are the conclusions drawn adequately supported by the results?

Partly

Are sufficient details of methods and analysis provided to allow replication by others?

Partly

Reviewer Expertise:

COVID-19 vaccination; health policies

I confirm that I have read this submission and believe that I have an appropriate level of expertise to confirm that it is of an acceptable scientific standard.

# Reviewer report: Reviewer response for version 1

**DOI:** 10.5256/f1000research.176950.r368449

**Published:** 2025-03-05

**Authors:** Gregory Barnsley

**Affiliations:** 1 London School of Hygiene and Tropical Medicine, London, UK

This report investigates the impact of COVID-19 vaccination on mortality in England between mid-2021 and mid-2023. The author observes that the age-stratified non-COVID mortality rates in the vaccinated population increase or remain stable over this period, whilst those in the unvaccinated population decrease. The author states that this observation is consistent with a vaccination-related decline in health. The author also observes periods where the COVID-19-related mortality rates in the unvaccinated are higher than those in the vaccinated, potentially showing a protective effect of vaccination against COVID-19-related disease. However, the author posits an alternative theory based on the unvaccinated population being generally more "unhealthy" (i.e. healthy vaccinee effect) as evidenced by higher rates of all-cause and non-covid related mortality in the unvaccinated population at the study start. The author claims that their approach can adjust for unobserved variables that explain the differences in health between the two comparison groups.

 The author has mixed his methods/reasoning into the report's introduction and results sections. It would be better to explore the approach in the methods section and highlight any potential limitations. The results should describe any major observations and the theorising should be limited to the discussion. Alternatively, the author could be more explicit about the theories he wants to test in the methods section; either way, the presentation should be improved. In Figure 3, the author should highlight how this relates to the other figures by overlaying the data or plotting on the same time scale.

 A third of the methods section describes how the author converted the ONS's age-stratified mortality rates (per 10000 person-years) to "mortality probability." The author should know that this process does not calculate a probability and rescales the given mortality rates. It is the equivalent of dividing the age-stratified mortality rates by 12*10000, calculating the age-stratified mortality rate per person-month. The report should compare the ONS rates as these are already at a more sensible scale.

 The report should also consider explicitly how the ONS definition of COVID-19-related death would impact these results. Excluding ICD10 codes U09.9 and U10.9 as COVID-related could bias these findings. The author should clearly explain the reasoning around how the assumption that *COVID-19 vaccination does not prevent non-COVID-19 deaths *supports the theory that *the difference in COVID-19 death rates (between unvaccinated and vaccinated) is explainable by inferior health at the onset*.

 The report does not sufficiently consider alternative explanations for the observed data. While the healthy-vaccinee effect might be strong in clinical trials (since these tend to recruit healthy volunteers), this effect might not be so strong in mass vaccination campaigns, particularly ones like COVID-19 that specifically target vulnerable populations. If we assume that this effect does explain the initial difference in non-COVID-19 mortality rates and that many of the unvaccinated (but not all, i.e. the vaccine-hesitant) are acutely ill, then we would expect to see a trend towards parity in the non-covid mortality rates of the two. As the acutely ill expire (or recover and get vaccinated), the mortality rates in the non-vaccinated would reduce in future months. This trend would be strong if the vulnerable and very elderly were targeted first for vaccination as they are at higher risk of becoming ill later (thus contributing to the mortality rate in the vaccinated population). This is to say nothing about the countless other confounding variables that could explain temporal differences in mortality across these groups, such as different temporal vaccine uptake in different ethnic or SES groups and different rates of adherence to restrictions.

 These alternative theories do not disprove the theory put forward in this report. However, they highlight that the methodology here cannot convincingly adjust for the potential health differences between the two comparison groups. While improper adjustment for confounding can increase bias, that is no excuse to ignore potential confounding. This report must focus on the actual observation it is theorising around (i.e. a decrease in the non-covid health gap between the vaccinated and the unvaccinated) and more convincingly explore/counter alternative explanations and consider sensitivities to their results.

 In conclusion, this report needs considerable reworking regarding its statistical and epidemiological content.

Is the work clearly and accurately presented and does it cite the current literature?

Partly

If applicable, is the statistical analysis and its interpretation appropriate?

No

Are all the source data underlying the results available to ensure full reproducibility?

Yes

Is the study design appropriate and is the work technically sound?

Partly

Are the conclusions drawn adequately supported by the results?

No

Are sufficient details of methods and analysis provided to allow replication by others?

Yes

Reviewer Expertise:

Epidemiology and mathematic modelling. I am not a demographer so I cannot comment on any particularities of looking at mortality rates.

I confirm that I have read this submission and believe that I have an appropriate level of expertise to state that I do not consider it to be of an acceptable scientific standard, for reasons outlined above.

# Response

**Published:** 2025-03-14

**Authors:** Jarle Aarstad

**Affiliations:** Western Norway University of Applied Sciences, Norway

Dear Referee 2,

 I highly appreciate your time and efforts in giving constructive feedback on my previous version of the paper. In the following, you will read how I have addressed your comments. For your information, I have also made minor corrections and editions in the text to improve accuracy and readability. Hopefully, the revised version will be uploaded shortly.  

 I look forward to hearing from you.

 Sincerely,

 The author.

**This report investigates the impact of COVID-19 vaccination on mortality in England between mid-2021 and mid-2023. The author observes that the age-stratified non-COVID mortality rates in the vaccinated population increase or remain stable over this period, whilst those in the unvaccinated population decrease. The author states that this observation is consistent with a vaccination-related decline in health. The author also observes periods where the COVID-19-related mortality rates in the unvaccinated are higher than those in the vaccinated, potentially showing a protective effect of vaccination against COVID-19-related disease. However, the author posits an alternative theory based on the unvaccinated population being generally more "unhealthy" (i.e. healthy vaccinee effect) as evidenced by higher rates of all-cause and non-covid related mortality in the unvaccinated population at the study start. The author claims that their approach can adjust for unobserved variables that explain the differences in health between the two comparison groups.**

Response: Below, I will address the particular issues you have raised in detail.

**The author has mixed his methods/reasoning into the report's introduction and results sections. It would be better to explore the approach in the methods section and highlight any potential limitations.**

Response: I agree with you, and in the revised version, I have removed the methodological approach from the Introduction, but mention the following: “To address the research gap [explained above in the Introduction], using English data covering 26 months from Apr 21 to May 23, 5 I elaborate an achievable approach by comparing all-cause mortality among COVID-19 vaccinated and unvaccinated with mortality not involving COVID-19. In the Methods section, I explain it in full detail.”

 Also, I highlight more extensively the potential limitations of the approach in the latter part of the Discussion, writing as follows: “The validity of the finding that vaccinated had significant protection between July 21 and Jan 22 hinges on non-systematic skewness in classifying false positives concerning mortality involving COVID-19 and false negatives concerning mortality not involving COVID-19. A relevant issue in this regard is that the English data excluded ICD10 death certificate codes U09.9 (Post-COVID condition, where the acute COVID had ended before the condition immediately causing death occurred) and U10.9 (Multisystem inflammatory syndrome associated with COVID-19) as criteria when classifying mortality involving COVID-19, but as this was the case only when the U07.1 (COVID-19, virus identified) or U07.2 (COVID-19, virus not identified) were *not* mentioned, I cannot see substantial skewness in false positives and negatives between vaccinated and unvaccinated. The potential limitation may nonetheless induce cautiousness when interpreting the data, which I encourage future research to address. The validity of the finding that vaccinated had non-significant protection from Feb 22 also has limitations, as relatively low mortality involving COVID-19 can be an alternative explanation. However, in Note [3], I elaborate extensively on the issue, concluding that the alternative explanation is not very likely, but I nonetheless encourage cautiousness when interpreting the data.”

 Please note that the revised text is an extension and further elaboration of the previous text addressing limitations.

 Please also see #4, which addresses revisions I have carried out in the Introduction by following advice from the other referee.

**The results should describe any major observations and the theorising should be limited to the discussion. Alternatively, the author could be more explicit about the theories he wants to test in the methods section; either way, the presentation should be improved.**

Response: In the revision, I have added a paragraph at the end of the Methods section where I argue in detail how distinctions between all-cause mortality and mortality not involving COVID-19 among vaccinated and unvaccinated, absent of control variables in populations with potentially different health statuses at the outset, can assess eventually genuine health effects. Please see #18. I agree with the referee that extensive discussions of empirical findings should not be conducted in the Results section, but presenting them without any interpretation will make it more difficult for the reader to interpret the text, I argue. Therefore, I point to findings, and briefly explain their potential meaning. In the revision, I have excluded some figures and included them in the Notes section (please see #6). As such, I have aimed to reduce the complexity of presenting the data and hope that the results are more interpretable. Also, a couple of places in the Results section, I refer to my explanation at the end of the Methods section.

**In Figure 3, the author should highlight how this relates to the other figures by overlaying the data or plotting on the same time scale.**

Response: I agree with your point, but unfortunately, it is challenging to carry out as the time scales are different; the English data I apply in my study use monthly observations, while the Our World in Data uses weekly ones. I find it challenging to convert the different time scales into one, as there is no distinct overlap in weekly and monthly observations. Moreover, in the revision, I have edited the text in the Results section and Abstract writing, “Further strengthening the interpretation was the relatively high mortality not involving COVID-19 among the vaccinated, corresponding with excess mortality during much of the same period”, as it more precisely reflects the genuine interpretation of the data.  

A third of the methods section describes how the author converted the ONS's age-stratified mortality rates (per 10000 person-years) to "mortality probability." The author should know that this process does not calculate a probability and rescales the given mortality rates. It is the equivalent of dividing the age-stratified mortality rates by 12*10000, calculating the age-stratified mortality rate per person-month. The report should compare the ONS rates as these are already at a more sensible scale.

Response: In the revision, I use the term monthly mortality rate per 100,000. (Of course, I could have used a yearly rate, but in my opinion, a monthly rate is more logical in the current context since I analyze monthly data.) I carry out the exercise, as I do, to assess how many died or survived of a population in a given month, vaccinated or unvaccinated, to estimate as statistically correct standard errors as possible using logistic regression.

**The report should also consider explicitly how the ONS definition of COVID-19-related death would impact these results. Excluding ICD10 codes U09.9 and U10.9 as COVID-related could bias these findings.**

Response: Thanks for this comment. In the revised version, I address the issue in the revision writing as follows: “The validity of the finding that vaccinated had significant protection between July 21 and Jan 22 hinges on non-systematic skewness in classifying false positives concerning mortality involving COVID-19 and false negatives concerning mortality not involving COVID-19. A relevant issue in this regard is that the English data excluded ICD10 death certificate codes U09.9 (Post-COVID condition, where the acute COVID had ended before the condition immediately causing death occurred) and U10.9 (Multisystem inflammatory syndrome associated with COVID-19) as criteria when classifying mortality involving COVID-19, but as this was the case only when the U07.1 (COVID-19, virus identified) or U07.2 (COVID-19, virus not identified) were *not* mentioned, I cannot see substantial skewness in false positives and negatives between vaccinated and unvaccinated. The potential limitation may nonetheless induce cautiousness when interpreting the data, which I encourage future research to address.”

**The author should clearly explain the reasoning around how the assumption that *COVID-19 vaccination does not prevent non-COVID-19 deaths *supports the theory that *the difference in COVID-19 death rates (between unvaccinated and vaccinated) is explainable by inferior health at the onset*.**

Response: At the end of the Methods section, I write as follows in the revision: “Assuming a 60% higher all-cause mortality rate among unvaccinated compared to vaccinated, in the absence of other information, can have two explanations: (i) the unvaccinated have inferior health at the outset compared to the vaccinated or (ii) vaccination protects against mortality. In addition, there can be a combination of (i) and (ii). If the mortality not involving COVID-19 is also 60% higher among unvaccinated, explanation (i) has more validity. The reason is that COVID-19 vaccination unlikely protects against mortality not involving COVID-19. ^16^ Conversely, if the mortality rate not involving COVID-19 is equal between unvaccinated and vaccinated, explanation (ii) has higher validity. The reason is that there is no other likely explanation than a vaccine effect as to why the all-cause mortality among unvaccinated compared to unvaccinated is higher than the mortality not involving COVID-19. Finally, if the mortality not involving COVID-19 is 20% higher among unvaccinated compared to the vaccinated, a combination of explanations (i) and (ii) has more validity. I.e., 20% higher mortality not involving COVID-19 among unvaccinated can be explained as inferior health status at the outset, while vaccination protection can explain 33% higher mortality among unvaccinated (((1.6/1.2)-1)*100). The explanations hinge on the assumption of non-systematic skewness in classifying false positives concerning mortality involving COVID-19 and false negatives concerning mortality not involving COVID-19, which I address in the Discussion. Further, the explanations hinge on the assumption that the mortality involving COVID-19 is not zero, which I address in Note 3.”

**The report does not sufficiently consider alternative explanations for the observed data. While the healthy-vaccinee effect might be strong in clinical trials (since these tend to recruit healthy volunteers), this effect might not be so strong in mass vaccination campaigns, particularly ones like COVID-19 that specifically target vulnerable populations. If we assume that this effect does explain the initial difference in non-COVID-19 mortality rates and that many of the unvaccinated (but not all, i.e. the vaccine-hesitant) are acutely ill, then we would expect to see a trend towards parity in the non-covid mortality rates of the two. As the acutely ill expire (or recover and get vaccinated), the mortality rates in the non-vaccinated would reduce in future months. This trend would be strong if the vulnerable and very elderly were targeted first for vaccination as they are at higher risk of becoming ill later (thus contributing to the mortality rate in the vaccinated population). This is to say nothing about the countless other confounding variables that could explain temporal differences in mortality across these groups, such as different temporal vaccine uptake in different ethnic or SES groups and different rates of adherence to restrictions. These alternative theories do not disprove the theory put forward in this report. However, they highlight that the methodology here cannot convincingly adjust for the potential health differences between the two comparison groups. While improper adjustment for confounding can increase bias, that is no excuse to ignore potential confounding. This report must focus on the actual observation it is theorising around (i.e. a decrease in the non-covid health gap between the vaccinated and the unvaccinated) and more convincingly explore/counter alternative explanations and consider sensitivities to their results.**

Response: You mention that “[w]hile the healthy-vaccinee effect might be strong in clinical trials (since these tend to recruit healthy volunteers), this effect might not be so strong in mass vaccination campaigns, particularly ones like COVID-19 that specifically target vulnerable populations.” Considering that statement, I cannot see that it aligns with the UK Office for National Statistics stating that “rates for COVID-19 unvaccinated adults in England “were higher for Black Caribbean, Black African and White Other ethnic groups. Rates were also higher for those living in deprived areas, who have never worked or are long-term unemployed, who are limited a lot by a disability, … or who are male.” Nor does it align with vaccine hesitancy (to which I refer in the revision), and Norwegian data showing much higher mortality among young unvaccinated in a population where practically zero young people died of COVID-19. Also, in the revised version, I explain in detail why vaccination cannot explain the difference in mortality not involving COVID-19. From my reading off the comment, it seems that the referee points to the dynamic of the group of people being transferred from the group of unvaccinated to the group of vaccinated during the time studied. That is definitely a relevant issue, which I have addressed in the Discussion, writing as follows (the text in the paper includes relevant references): “During the study period, a share of people in the unvaccinated group were transferred to the vaccinated. Assuming they had inferior health status at the outset, it may explain the relative increase (decrease) in mortality among the vaccinated (unvaccinated). However, those who remained unvaccinated, on the contrary, had inferior health status at the outset, making the above reasoning implausible. Ceteris paribus, one may even oppositely conclude that it would decrease (increase) relative mortality among vaccinated (unvaccinated). (In Note 7, I add: “People in England under 70 years old but clinically extremely vulnerable were prioritized vaccination with those aged between 70-74. Hence, they were prioritized early.”) Since most elderly candidates had been offered vaccine before Apr 21, I nonetheless assume the estimates were not substantially skewed over the study period, as relatively few people die in younger age cohorts.”

**In conclusion, this report needs considerable reworking regarding its statistical and epidemiological content.**

Response: Above, you will read how I have addressed the issues raised.

# Reviewer report: Reviewer response for version 1

**DOI:** 10.5256/f1000research.176950.r363090

**Published:** 2025-02-13

**Authors:** Mario Coccia

**Affiliations:** 1 Consiglio Nazionale delle Ricerche Area di Ricerca di Torino, Turin, Piedmont, Italy

The temporal protection and declining health of the COVID-19 vaccinated in England: A 26-month comparison of the mortality involving and not involving COVID-19 among vaccinated vs. unvaccinated

 The topics of this paper is interesting but the structure and content must be revised, and results have to be  explained by authors.

 Title has to be shorter, indicating the period under study. 

 Abstract has to clarify the goal and health policy implications to face the next pandemics similar to COVID-19.

 Introduction has to better clarify the research questions of this study, indicating the gap presents in literature that this study endeavors to cover,  and provide more theoretical background about these topics. After that authors can focus on the topics of this study to provide a correct analysis for fruitful discussion (See suggested readings that must be all read and used in the text). 

 The methods of this study is not clear. The section of Materials and methods must be re-structured with the following three sections:

 •    Sample and data

 •    Measures of variables

 •    Models and Data analysis procedure. 

 Results. 

 Figure 1 and 2 are not clear for readers. First clarify the measure on y-axis. Second I suggest merging some of them. The legend is not clear and has to be put for all graphs. Lines are better than dots, using continuous vs. dotted lines for vaccinated vs. unvaccinated. In Figure 1, C1 and C2 have the same title…

 Insert a vertical line in figures to divide the COVID and post-covid period to be clear.

 Frankly these figures are messy. Do other better and clearer otherwise the information about results are useless. 

 The paper has a lot of figures/graphs (in Figure 1 and 2) that are difficult to digest, some of them can be put in appendix and inserting in the text the most important ones to improve the readability…

 Discussion. 

 First, authors have to synthesize the main results in a simple table to be clear for readers and then show what this study adds compared to other studies. 

 Although the Results section provides a detailed description of the data collected and analyzed, there needs to be a more critical synthesis and comparison of the findings with the literature. Better comment on whether the results were expected for each set of findings; go into greater depth to explain unexpected findings. If appropriate, note any unusual or unanticipated patterns or trends that emerged from your results (directly in figures) and explain their meaning concerning the research problem under study here.

 Unvaccinated have higher all causes of mortality during the COVID-19 because there was some restrictions to make diagnostics or to have access to hospitals?   

 Moreover, the higher mortality of vaccinated can be due to the effects of vaccines on immune system that has created some disorder to face diseases. 

 Moreover, either compare your results with the findings from other studies or use the studies to support results. Insert a claim for how the results can be applied more generally, beyond England. 

 Authors have to describe lessons learned, proposing recommendations that can help improve a next pandemic crises, or highlighting best practices.

 The conclusion is better as an autonomous section. Conclusion has not to be a summary, but authors have to focus on manifold limitation. In addition, now the Conclusion does not adequately discuss the theoretical and managerial implications of the study. Discuss better how a gap in literature has been addressed. Make sure you clarify: 1) Theoretical Implications, 2) Policy Implications based on health systems improvement and good governance to face next emergencies, and 3) Future Research.

 Overall, then, the paper is interesting, but Theoretical framework is weak, and some results create confusion… structure of the paper has to be improved; study design, discussion and presentation of results have to be clarified using suggested comments.

 Suggested readings of relevant papers that have to be read and used to improve the paper.

 Harrison, C.,et al., 2024 ^1^   

 Meyer, C.et al., 2023. ^2^   

 Coccia M. 2023. ^3^

 Halford, F., et al., 2024. ^4^    

 Coccia, M. and Benati, I. (2024), ^5^

 Griggs, E.P., et., 2024. ^6^  

 Mink, S., et al., 2024. ^7^    

 Coccia M. 2022. ^8^

 Jones, R.P., Ponomarenko, A. 2023. ^9^    

 Kirwan, P.D., et al., 2022. ^10^    

 Wekking, D., et al., 2024. ^11^

Is the work clearly and accurately presented and does it cite the current literature?

Partly

If applicable, is the statistical analysis and its interpretation appropriate?

Partly

Are all the source data underlying the results available to ensure full reproducibility?

Partly

Is the study design appropriate and is the work technically sound?

Partly

Are the conclusions drawn adequately supported by the results?

Partly

Are sufficient details of methods and analysis provided to allow replication by others?

Partly

Reviewer Expertise:

COVID-19 vaccination; health policies

I confirm that I have read this submission and believe that I have an appropriate level of expertise to state that I do not consider it to be of an acceptable scientific standard, for reasons outlined above.

## References

[1] : The impact of COVID-19 vaccination on patients with congenital heart disease in England: a case-control study. *Heart* .2024;110(23) : 10.1136/heartjnl-2024-324470 1372-1380 10.1136/heartjnl-2024-324470 39393905 PMC11671934

[2] : Using the precaution adoption process model to understand decision-making about the COVID-19 booster vaccine in England. *Vaccine* .2023;41(15) : 10.1016/j.vaccine.2023.02.047 2466-2475 10.1016/j.vaccine.2023.02.047 36933983 PMC9935297

[3] : Sources, diffusion and prediction in COVID-19 pandemic: lessons learned to face next health emergency. *AIMS Public Health* .2023;10(1) : 10.3934/publichealth.2023012 145-168 10.3934/publichealth.2023012 37063362 PMC10091135

[4] : Temporal changes to adult case fatality risk of COVID-19 after vaccination in England between May 2020 and February 2022: a national surveillance study. *J R Soc Med* .2024;117(6) : 10.1177/01410768231216332 202-211 10.1177/01410768231216332 38096898 PMC11299124

[5] : Effective health systems facing pandemic crisis: lessons from COVID-19 in Europe for next emergencies. *International Journal of Health Governance* .2024;29(2) : 10.1108/IJHG-02-2024-0013 89-111 10.1108/IJHG-02-2024-0013

[6] : Clinical Epidemiology and Risk Factors for Critical Outcomes Among Vaccinated and Unvaccinated Adults Hospitalized With COVID-19-VISION Network, 10 States, June 2021-March 2023. *Clin Infect Dis* .2024;78(2) : 10.1093/cid/ciad505 338-348 10.1093/cid/ciad505 37633258 PMC11293024

[7] : Antibody levels versus vaccination status in the outcome of older adults with COVID-19. *JCI Insight* .2024;9(20) : 10.1172/jci.insight.183913 10.1172/jci.insight.183913 39435658 PMC11529978

[8] : COVID-19 Vaccination is not a Sufficient Public Policy to face Crisis Management of next Pandemic Threats. *Public Organization Review* .2023;23(4) : 10.1007/s11115-022-00661-6 1353-1367 10.1007/s11115-022-00661-6

[9] : COVID-19-Related Age Profiles for SARS-CoV-2 Variants in England and Wales and States of the USA (2020 to 2022): Impact on All-Cause Mortality. *Infect Dis Rep* .2023;15(5) : 10.3390/idr15050058 600-634 10.3390/idr15050058 37888139 PMC10606787

[10] : Trends in COVID-19 hospital outcomes in England before and after vaccine introduction, a cohort study. *Nat Commun* .2022;13(1) : 10.1038/s41467-022-32458-y 4834 10.1038/s41467-022-32458-y 35977938 PMC9382625

[11] : The impact of COVID-19 on cancer patients. *Cytokine Growth Factor Rev* .2024;75: 10.1016/j.cytogfr.2023.11.004 110-118 10.1016/j.cytogfr.2023.11.004 38103990

# Response

**Published:** 2025-03-14

**Authors:** Jarle Aarstad

**Affiliations:** Western Norway University of Applied Sciences, Norway

Dear Referee 1,

 I highly appreciate your time and efforts in giving constructive feedback on my previous version of the paper. In the following, you will read how I have addressed your comments. For your information, I have also made minor corrections and editions in the text to improve accuracy and readability. Hopefully, the revised version will be uploaded shortly.  

 I look forward to hearing from you.

 Sincerely,

 The author.

 1. The temporal protection and declining health of the COVID-19 vaccinated in England: A 26-month comparison of the mortality involving and not involving COVID-19 among vaccinated vs. unvaccinated

 The topics of this paper is interesting but the structure and content must be revised, and results have to be  explained by authors.

Response: Thanks for this overall positive feedback. Below, you will read how I have addressed each of your comments.

 2. Title has to be shorter, indicating the period under study. 

Response: In the revision, the title is shortened and indicates the period under study. It reads as follows: “Mortality involving and not involving COVID-19 among vaccinated vs. unvaccinated in England between Apr 21 and May 23”

 3. Abstract has to clarify the goal and health policy implications to face the next pandemics similar to COVID-19.

Response: In the revision, I added the following sentences at the end of the Abstract: “An implication of the study, which particularly has relevance for future pandemics, is that COVID-19 vaccinated may have a limited time window of protection and can even be exposed to detrimental health consequences. The pattern should be followed up over an extended period in future research. Also, future research should examine different age groups, vaccination types, and the number of doses given.”

 4. Introduction has to better clarify the research questions of this study, indicating the gap presents in literature that this study endeavors to cover, and provide more theoretical background about these topics. After that authors can focus on the topics of this study to provide a correct analysis for fruitful discussion (See suggested readings that must be all read and used in the text). 

Response: The Introduction has been substantially revised. The initial part of the first paragraph is largely unaltered, except that I address the concept of vaccine hesitancy and also include relevant references. The last sentences of the first paragraph, on the other hand, are novel, illustrating with Norwegian data that (1) COVID-19 vaccinated and unvaccinated have different health status at the outset and (2) including control variables can make estimates less, not more, accurate. I believe that the above issues better address the study’s theoretical background concerning previous relevant research and substantial argument. The second paragraph addresses the study’s research gap. Also, I explain there that I will empirically study English data covering 26 months from Apr 21 to May 23, but following your recommendation, I just briefly mention the methodological approach and emphasize that I will explain it in detail in the Methods section. In the third paragraph, I explicitly address the study’s research question and major contribution. In the Introduction’s final paragraph, I added more references concerning the literature on COVID-19 vaccination and outcomes. Finally, I conclude the Introduction by stating the following: “Applying my approach to the English data, I particularly contribute to the research on the link between COVID-19 vaccination and mortality, as most previous studies have been carried out in non-randomized contexts and, accordingly, even in the presence of control variables, exposed to challenges concerning validity addressed above.”

 5. The methods of this study is not clear. The section of Materials and methods must be re-structured with the following three sections:

 •    Sample and data

 •    Measures of variables

 •    Models and Data analysis procedure. 

Response: In the revision, I have followed your recommendation. The new subsections include extended and hopefully substantially improved information about the study’s methodology concerning the requested issues. Please also see #16 and #18.

 6. Results. 

 Figure 1 and 2 are not clear for readers. First clarify the measure on y-axis. Second I suggest merging some of them. The legend is not clear and has to be put for all graphs. Lines are better than dots, using continuous vs. dotted lines for vaccinated vs. unvaccinated. In Figure 1, C1 and C2 have the same title…

 Insert a vertical line in figures to divide the COVID and post-covid period to be clear.

 Frankly these figures are messy. Do other better and clearer otherwise the information about results are useless. 

 The paper has a lot of figures/graphs (in Figure 1 and 2) that are difficult to digest, some of them can be put in appendix and inserting in the text the most important ones to improve the readability…

Response: In the revision, I have followed your suggestions. All figures now include explanations of the vertical axes. Also, I have moved Figures 1 C1 and C2 to the Notes section (in the revision, they are part of Figure 5). (Figures 1 C1 and C2 had the same title because they were identical, except for different scaling.) Similarly, I have added Figure 2C to the Notes section. In the revision, it is Figure 6. Finally, Figure 2D is a separate figure in the revision, named Figure 3.

 Concerning legends, I have done my utmost to use them as a tool to maximize graph readability.

 You note that lines are better than dots. I would agree if the observations were linear, but since I study months as dummy observations, I find it more correct to include them as dots. Also, from my experience, it is normal to include observations as dots in other studies when dealing with time periods. Independent of opinion, I argue that the new figures are clearer to read as they are larger, particularly on the vertical axes.

 You note that I should include a vertical line in the figures “to divide the COVID and post-covid period”, but all months in the data include the COVID period.

 7.   Discussion. 

 First, authors have to synthesize the main results in a simple table to be clear for readers and then show what this study adds compared to other studies. 

 Although the Results section provides a detailed description of the data collected and analyzed, there needs to be a more critical synthesis and comparison of the findings with the literature. Better comment on whether the results were expected for each set of findings; go into greater depth to explain unexpected findings. If appropriate, note any unusual or unanticipated patterns or trends that emerged from your results (directly in figures) and explain their meaning concerning the research problem under study here. Unvaccinated have higher all causes of mortality during the COVID-19 because there was some restrictions to make diagnostics or to have access to hospitals?   

 Moreover, the higher mortality of vaccinated can be due to the effects of vaccines on immune system that has created some disorder to face diseases. 

Response: The first part of the Discussion has been edited a lot. First, I address the study’s core finding. Then, I address how they contribute to and align with the research literature.

 Concerning “a more critical synthesis and comparison of the findings with the literature” and the assessment of “deeper findings” I argue in the revision that “the study’s perhaps major contribution was to elaborate a useful tool to compare non-randomized groups in the absence of control variables, which even in their presence can even make statistical conclusions less, not more, accurate. Thus, as most previous studies on the link between COVID-19 vaccination and mortality have been carried out in non-randomized contexts and, accordingly, even in the presence of control variables exposed to challenges concerning validity, this study has illustrated and applied a useful tool to address those limitations. Moreover, I argue that the tool has general applicability as it can also be used in other research contexts.”

 Concerning “unexpected” findings, I do not address the topic explicitly but emphasize that my approach has concluded that the vaccine likely has had a temporal but declining effect. Also, I show how the effect in the long term can be detrimental. These different findings align with the established research literature.

 You write that “Unvaccinated have higher all causes of mortality during the COVID-19 because there was some restrictions to make diagnostics or to have access to hospitals?” That may be a possibility, but if yes, it aligns with the non-randomized difference between vaccinated and unvaccinated, which this study has emphasized in particular.

 Finally, you write that “the higher mortality of vaccinated can be due to the effects of vaccines on immune system that has created some disorder to face diseases”, and I agree with you. However, in line with your comment I refer to studies indicating that the vaccine can have adverse effects, but addressing your issue in detail, I argue is beyond the scope of the study.

 8. Moreover, either compare your results with the findings from other studies or use the studies to support results. Insert a claim for how the results can be applied more generally, beyond England. 

 Authors have to describe lessons learned, proposing recommendations that can help improve a next pandemic crises, or highlighting best practices.

Response: I argue that the Discussion should address findings and contributions. Going very much more into detail by adding new research streams, I believe would increase the complexity and perhaps even blur my main objective for carrying out the analyses as I did. However, I have added a new section, “Implications”, to address some of your issues and refer to your suggested studies.

 9. The conclusion is better as an autonomous section. Conclusion has not to be a summary, but authors have to focus on manifold limitation. In addition, now the Conclusion does not adequately discuss the theoretical and managerial implications of the study. Discuss better how a gap in literature has been addressed. Make sure you clarify: 1) Theoretical Implications, 2) Policy Implications based on health systems improvement and good governance to face next emergencies, and 3) Future Research.

Response: I believe the revised Discussion better addresses the issues the referee has raised.

 10. Overall, then, the paper is interesting, but Theoretical framework is weak, and some results create confusion… structure of the paper has to be improved; study design, discussion and presentation of results have to be clarified using suggested comments.

Response: I hope and believe that my revisions, which I have commented on elsewhere in this referee report, have improved the paper concerning theoretical framework, structure, study design, and the presentation of results.

 11. Suggested readings of relevant papers that have to be read and used to improve the paper.

 Harrison, C.,et al., 2024 ^1^

 Meyer, C.et al., 2023. ^2^

 Coccia M. 2023. ^3^

 Halford, F., et al., 2024. ^4^

 Coccia, M. and Benati, I. (2024), ^5^

 Griggs, E.P., et., 2024. ^6^

 Mink, S., et al., 2024. ^7^

 Coccia M. 2022. ^8^

 Jones, R.P., Ponomarenko, A. 2023. ^9^

 Kirwan, P.D., et al., 2022. ^10^

 Wekking, D., et al., 2024. ^11^

Response: In the revision, I incorporate your suggested references (in addition to other references) as follows: “COVID-19 vaccination has been recommended to most population groups, including people with comorbidities (Wekking et al., 2024). Studies have further indicated that COVID-19 vaccination can prevent mortality (Halford et al., 2023; Harrison et al., 2024; Kirwan et al., 2022), but along with research showing that antibody levels were a superior predictor (Mink et al., 2024), the effect declines, and research has even shown ‘a positive correlation between people fully vaccinated and COVID-19 mortality’ (Coccia, 2023a, p. 1353).” I refer to Meyer, C.et al. (plus another reference) in the following sentence (at the beginning of the Introduction): “The statement aligns with vaccine hesitancy research (Lamot & Kirbiš, 2024; Meyer et al., 2023) and further indicates that unvaccinated have inferior health at the outset compared to vaccinated, inducing biased comparisons as the groups are not randomly assigned.” I refer to Jones, R.P., Ponomarenko, A. 2023 in Note 6, writing as follows: “For an extensive review of all-cause mortality in England and Wales, please see Jones and Ponomarenko (2023).”

 Concerning the incorporated references to Coccia M. 2022, Coccia, M. and Benati, I. (2024), and Griggs, E.P., et., 2024, please see #8.
